# Azithromycin Treatment vs Placebo in Children With Respiratory Syncytial Virus–Induced Respiratory Failure: A Phase 2 Randomized Clinical Trial

**DOI:** 10.1001/jamanetworkopen.2020.3482

**Published:** 2020-04-23

**Authors:** Michele Kong, Wei Wei Zhang, Kate Sewell, Gregory Gorman, Hui-Chien Kuo, Inmaculada Aban, Namasivayam Ambalavanan, Richard J. Whitley

**Affiliations:** 1 Department of Pediatrics, University of Alabama at Birmingham; 2 McWhorter School of Pharmacy, Samford University, Birmingham, Alabama

## Abstract

**Importance:**

Despite a high disease burden, there is no effective treatment for respiratory syncytial virus (RSV) infection.

**Objectives:**

To determine whether administration of azithromycin (AZM) to children with RSV-induced respiratory failure is safe and to define the effect of AZM therapy on nasal matrix metalloproteinase 9 (MMP-9) levels.

**Design, Setting, and Participants:**

This randomized, double-blind, placebo-controlled phase 2 trial was conducted at a single tertiary pediatric intensive care unit from February 2016 to February 2019. The study included children with RSV infection who were admitted to the pediatric intensive care unit and required respiratory support via positive pressure ventilation (invasive and noninvasive). A total of 147 children were screened; 90 were excluded for not meeting inclusion criteria, having an absent legal guardian, lacking pharmacy support, or having a language barrier and 9 declined participation, resulting in 48 patients enrolled in the study.

**Intervention:**

Receipt of standard dose AZM (10 mg/kg/d), high-dose AZM (20 mg/kg/d), or a matching placebo of normal saline intravenously for 3 days.

**Main Outcomes and Measures:**

Nasal and endotracheal samples were collected at baseline as well as at 24 hours and 48 hours after start of treatment. The secondary outcome was to determine treatment effect on clinical outcome measures, including days of positive pressure ventilation and length of hospital stay.

**Results:**

A total of 48 patients were enrolled in the trial, with a median (range) age at randomization of 12 (1 to 125) months; 36 participants (75.0%) were younger than 2 years. Overall, 26 participants (54.2%) were boys, and 29 (60.4%) had a comorbidity. A total of 16 patients were randomized into each trial group (ie, placebo, standard-dose AZM, and high-dose AZM). Baseline demographic characteristics were comparable among the 3 groups. Both doses of AZM were safe, with no adverse events observed. No difference in nasal MMP-9 levels were observed between treatment groups. Among those who required mechanical ventilation and received high-dose AZM, endotracheal active and total MMP-9 levels were lower on day 3. Compared with baseline, active and total MMP-9 levels in endotracheal aspirates were 1.0 log lower in the high-dose AZM group (active MMP-9: 99.8% CI, −1.28 to −0.64; *P* < .001; total MMP-9: 99.8% CI, −1.37 to −0.57; *P* < .001). Patients who received high-dose AZM had fewer median (interquartile range) hospital days compared with those receiving the placebo (8 [6-14] days vs 11 [8-20] days; mean ratio estimate, 0.57; 95% CI, 0.38-0.87; *P* = .01).

**Conclusions and Relevance:**

In this phase 2 randomized clinical trial, both doses of AZM were safe. While nasal MMP-9 levels were unchanged among treatment groups, endotracheal MMP-9 levels were lower among those who received high-dose AZM. The positive secondary clinical outcome, while exploratory, provides insight for end points in a multicenter randomized trial.

**Trial Registration:**

ClinicalTrials.gov Identifier: NCT02707523

## Key Points

**Question:**

Is azithromycin (AZM) safe, and does it reduce nasal and endotracheal matrix metalloproteinase 9 (MMP-9) levels in children with respiratory syncytial virus–induced respiratory failure?

**Findings:**

In this randomized phase 2 clinical trial that included 48 children admitted to the pediatric intensive care unit with respiratory syncytial virus lung disease who required positive pressure ventilation, AZM was safe. No difference in nasal MMP-9 levels was observed between treatment group, but in those who required mechanical ventilation and received a high dose of AZM, endotracheal active and total MMP-9 levels were lower on day 3 of treatment.

**Meaning:**

In this study, high doses of AZM were safe, reduced endotracheal MMP-9 levels in patients receiving mechanical ventilation, and potentially improved outcomes in critically ill children with respiratory syncytial virus infections.

## Teaser

This phase 2 randomized clinical trial investigates the safety of azithromycin for children with respiratory syncytial virus–induced respiratory failure and assesses the effect of azithromycin therapy on nasal matrix metalloproteinase 9 levels.

## Introduction

Respiratory syncytial virus (RSV) infection has a substantial global disease burden.^1,2,3,4^ Matrix metalloproteinases (MMPs) are proteases implicated in the pathogenesis of both acute and chronic lung disease.^5,6,7,8,9,10,11^ In 2015, we reported^12^ that early elevation of endotracheal MMP-9 levels was positively associated with RSV disease severity and that MMP-9 inhibition decreased RSV replication in a murine model, suggesting that MMP-9 might be a potential marker of disease severity and a therapeutic target in RSV disease.

Macrolides are a class of antibiotics that are known to penetrate cells, making them active against intracellular pathogens; azithromycin (AZM) is reported to have a long intracellular half-life.^13^ In addition, AZM is an immune modulator that has been reported to provide clinical benefit in inflammatory airway diseases.^14,15^ Therefore, we performed a randomized, placebo-controlled phase 2 trial to test the hypothesis that AZM therapy during RSV-induced respiratory failure was safe and would reduce nasal and lung MMP-9 levels as well as to explore preliminary effects on the disease course for planning a subsequent definitive trial.

## Methods

### Participants

Eligible participants included all children admitted to the pediatric intensive care unit (PICU) at Children’s of Alabama (Birmingham) with a diagnosis of RSV infection and requiring positive pressure ventilation, invasive or noninvasive, including bilevel positive airway pressure (BiPAP) or high-flow nasal cannula (HFNC) oxygen (ie, >1 L/kg/min of flow, with ≥5 L/min flow for children weighing <5 kg). When the trial began in February 2016, it only included patients with RSV with respiratory failure requiring mechanical ventilation, but in December 2016, it was expanded to include patients with respiratory failure supported on noninvasive positive pressure ventilation. This was to reflect the overall change in the PICU’s practice whereby more patients had a trial of management using BiPAP or HFNC before mechanical ventilation. Exclusion criteria included use of AZM within 7 days of PICU admission, any contraindication to AZM use, cardiac arrhythmias, electrocardiogram with QT interval corrected for heart rate of at least 450 milliseconds, history of pyloric stenosis, and receipt of immunosuppressant.

During hospitalization, all patients were treated according to the American Academy of Pediatrics guidelines for the management of bronchiolitis,^16^ primarily supportive care. The study protocol was approved by the University of Alabama at Birmingham institutional review board, and a data and safety monitoring board serially evaluated the study. An Investigational New Drug approval was obtained from the US Food and Drug Administration for the use of AZM in this trial (Investigational New Drug number, 127632). Participants’ legal guardians provided written informed consent before randomization into the trial. The Consolidated Standards of Reporting Trials (CONSORT) guideline was used for the reporting of this trial.^17^ The trial protocol is available in Supplement 1.

### Study Design and Treatment

The study was a double-masked, placebo-controlled parallel group trial with 3 treatment groups. All participants, their families, health care professionals, and study staff were masked to study medication allocation, with the exception of the biostatistician and the pharmacist. Participants were randomized according to a permuted-block design to receive either placebo (saline) or AZM (Fresenius Kabi) at 10 mg/kg/d (ie, standard dose) or 20 mg/kg/d (ie, high dose) intravenously every 24 hours for 3 days. The randomization schedule was created by the study biostatistician and provided to the study pharmacist for implementation.

Nasal and endotracheal samples (if intubated) were collected from all participants before treatment as well as at 24 hours and at 48 hours after treatment started. For patients who were intubated, only nasal samples were collected once extubated. No long-term follow-up was performed.

The study spanned 3.5 RSV seasons, from February 2016 to February 2019. Owing to the initial age restriction placed by the Food and Drug Administration, the first 9 participants enrolled were older than 6 months. Once this number was reached and no safety concerns were evident as determined by the data and safety monitoring board, infants younger than 6 months were eligible for enrollment in the study.

The primary outcomes of this trial were as follows: (1) to ascertain the safety of AZM and its pharmacokinetics and (2) to determine whether treatment with AZM resulted in decreased nasal MMP-9 levels. When the study protocol was expanded to include patients who were not ventilated, the primary outcome also changed from endotracheal MMP-9 levels to nasal MMP-9 levels. The secondary outcomes were to determine the in vivo efficacy of AZM administration on improving clinical outcome measures, specifically duration of ventilatory support and oxygenation as well as PICU and hospital lengths of stay.

### Nasal and Endotracheal Aspirate Collection and Processing

Nasal samples were collected using a nasal swab inserted through the nares. The swab was then immediately placed in a vial containing 3 mL of viral transport media (Quidel). Endotracheal aspirates were collected via endotracheal tube suctioning using an 8 French suction cannula (CareFusion) and centrifuged at 500*g* for 10 minutes to separate supernatant from cells and mucus (pellet). All samples were kept at 4 °C for further analysis.

### Assay of AZM and Urea Levels and Pharmacokinetic Analysis

We measured AZM and urea levels in endotracheal and plasma samples using liquid chromatography–tandem mass spectrometric assays. Dilution estimations of endotracheal samples were calculated using the urea dilution method.^18^ Peak plasma levels (C_max_) and time to C_max_ (T_max_) of AZM were determined by visual evaluation of the concentration vs time profile. The area under the curve (AUC) values were calculated using the trapezoidal rule. The half-life (t_1/2_) of AZM was calculated as natural log of 2 / k_e_, in which *k_e_* was the terminal elimination rate constant estimated by linear regression analysis of the terminal portion of the concentration-time profile. Penetration ratios for AZM in samples from the endotracheal compartment were calculated as the AUC of the endotracheal compartment divided by the AUC of plasma.

### Biologic Outcome Measurement

Measurement of active MMP-9, total MMP-9, and tissue inhibitor of metalloproteinase 1 (TIMP-1) were done using established fluorometric assays (F9M00 and DTM100, respectively; R&D Systems). Interleukin 1 (IL-1), IL-2, IL-4, IL-6, IL-8, IL-10, IL-12, IL-13, tumor necrosis factor α (TNF-α), and interferon γ (IFN-γ) were analyzed via electrochemiluminescence using the Meso Scale Discovery V-PLEX Cytokine Panel 1 Human Kit (K15049D-1; Meso Scale Diagnostics). We measured RSV loads by reverse-transcription quantitative polymerase chain reaction using known concentrations of RSV to derive a standard curve.^19^ Standards and negative controls were included and tested with each polymerase chain reaction assay. We reported RSV quantification as log_10_ copies per mL.

### Clinical Data Assessment

The clinical data collected included duration of respiratory support (focusing on days of mechanical ventilation, noninvasive positive pressure ventilation via BiPAP or HFNC, and total duration of supplemental oxygen), PICU stay, and hospital stay. Comorbidities and the presence of multiorgan failure^20^ were also identified.

### Sample Size Determination and Statistical Analysis

This study was designed to have a total sample of 48 participants divided in 3 groups, with 16 per group, to detect a 25% and 37.5% decrease in lung MMP-9 activity in the standard-dose and high-dose groups, respectively, relative to placebo, with 80% power using analysis of variance. The assumptions were based on MMP-9 levels found in endotracheal aspirate of children with RSV-induced respiratory failure.^13^

### Statistical Analysis

Means, standard deviations, medians, quartiles, and ranges (minimum, maximum) were used to describe continuous variables, and counts and percentages were used for categorical variables. To compare baseline characteristics among the treatment groups, we used the Kruskal-Wallis test for age, height, and weight due to evidence of nonnormality of the distribution of the values. We used either χ^2^ or Fisher exact test for categorical variables depending on expected cell counts.

We analyzed inflammatory markers by first performing log_10_ transformation to address the skewedness of the distribution and extreme outliers. We then calculated the difference at each point relative to baseline to obtain the change (ie, pretreatment and posttreatment) outcome and fitted a generalized linear mixed model with random intercepts and assumed unequal variances across treatment groups with change from baseline as the outcome. The Bonferroni approach was used to adjust for multiple testing (with a 2-tailed *P*  <  .002 as the threshold for statistical significance) and simultaneous confidence intervals (99.8% confidence level).

For clinical outcomes (measured in number of days), we used a generalized linear model assuming a negative binomial distribution using log link function to address possible overdispersion due to some extreme observations. For this set of analyses, a 2-tailed *P* < .05 was set as the level of significance, with 95% CIs. All analyses were done using SAS version 9.4 (SAS Institute).

## Results

### Enrollment and Baseline Characteristics

**Figure 1.**
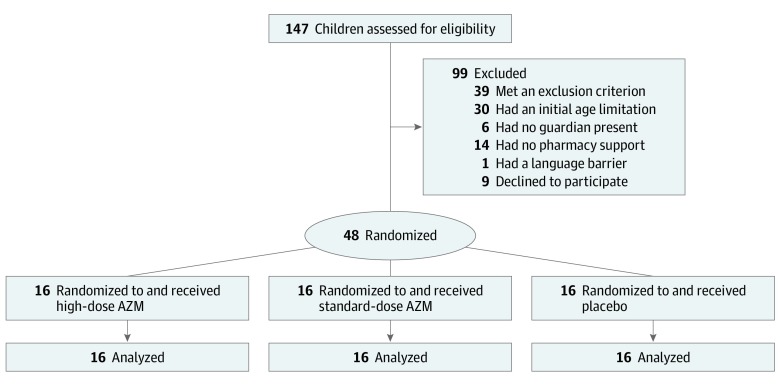
Flow Diagram of Trial AZM indicates azithromycin.

A total of 147 patients were screened, and 48 patients (32.7%) were randomized (Figure 1). All enrolled patients completed the study. The median (range) age of patients at randomization was 12 (1-125) months, with 36 (75.0%) younger than 2 years. Overall, 26 participants (54.2%) were boys, and 29 (60.4%) had a comorbidity. Baseline demographic characteristics were comparable among the 3 groups (eTable in Supplement 2). At the time of randomization, 34 patients (70.8%) required mechanical ventilation, 2 (4.2%) received BiPAP support, and the remaining 12 patients (25.0%) received HFNC. Once extubated, 4 (11.8%) received BiPAP, and 22 (64.8%) were transitioned to HFNC. A total of 5 of 34 patients (14.7%) were extubated before day 3. No patients who initially received BiPAP or HFNC at randomization required intubation.

### Pharmacokinetics of AZM

The pharmacokinetic parameters for AZM are listed in Table 1. A linear dose-dependent response was observed for the mean (SD) AUC values of AZM in plasma between the standard-dose and high-dose groups (14.8 [5.5] μg x h/mL vs 25.3 [13.2] μg x h/mL), while a nonlinear dose response was noted for the endotracheal samples between AZM groups (892.4 [383.7] μg x h/mL vs 807.3 [331.0] μg x h/mL). We observed that mean (SD) C_max_ values were higher in the high-dose group for plasma than in the standard-dose group (0.34 [0.06] μg/mL vs 0.22 [0.09] μg/mL; *P* < .001) and comparable for corresponding endotracheal samples (8.7 [1.1] μg/mL vs 11.0 [1.5] μg/mL; *P* = .008). The times to C_max_ were the same between dose groups for plasma and for endotracheal samples in the standard-dose group, but it was prolonged for the endotracheal samples in the high-dose group. An inverse dose-dependent penetration ratio (2:1) was observed in the endotracheal samples between the standard-dose and high-dose groups, respectively (48 h for the standard-dose group; 96 h for the high-dose group). This observation was due to comparable endotracheal AUC values between dose groups, while the plasma AUC values exhibited a linear dose dependent relationship. The comparable endotracheal AUC values were likely due to extensive distribution of AZM in the respiratory tract, resulting in drug saturation.

**Table 1.** Pharmacokinetic Parameters for Standard- and High-Dose AZM Using Urea-Corrected Data

Parameter	Mean (SD)			
	Receiving 10 mg/kg AZM		Receiving 20 mg/kg AZM	
	Plasma	Endotracheal	Plasma	Endotracheal
C_max_, μg/mL	0.22 (0.09)	11 (1.5)	0.34 (0.06)	8.7 (1.1)
T_max_, h	48	48	48	96
AUC, 0-144 h, μg × h/mL	14.8 (5.5)	892.4 (383.7)	25.3 (13.2)	807.3 (331)
AUC_endotracheal_ / AUC_plasma_	60.3		31.9	
Half-life, h	76 (17)	78 (31)	102 (33)	385 (63)

Abbreviations: AUC, area under the curve; AZM, azithromycin; C_max_, peak plasma levels; T_max_, time to peak plasma levels.

### Safety Profile and Adverse Events

All participants were monitored daily for adverse events using National Institutes of Health event recording^21^ from the time of study enrollment until discharge from the PICU. No gastrointestinal adverse events (ie, diarrhea, vomiting, or abdominal distention), feeding intolerance, thrush, intravenous site reactions, or rash were documented in any of the study participants. We measured QT interval corrected for heart rate daily during the treatment phase, and all were within normal range. There were no abnormal vital signs or arrhythmias noted during or within 4 hours of drug administration. Finally, no abnormal laboratory values attributable to the study drug were documented.

### Profiles of MMP-9 and Cytokine Levels at Baseline and After Treatment

**Figure 2.**
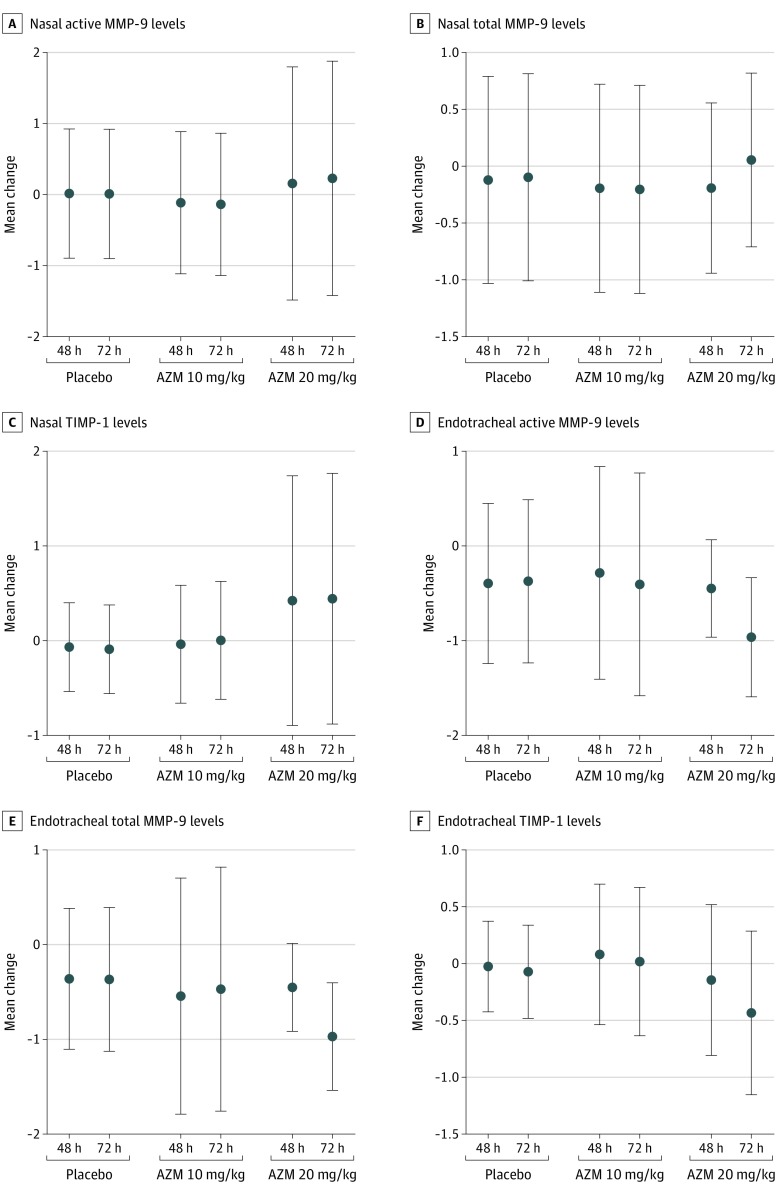
Change in MMP-9 and TIMP-1 Levels Each panel shows the estimate of means with 99.8% CIs, whereby treatment effect is defined as the change (ie, posttreatment minus pretreatment) at each day for a given treatment. Therefore, a nonzero mean difference indicates a treatment effect for the given day and treatment (placebo vs standard-dose azithromycin [AZM] vs high-dose AZM) for the cytokines measured in the nasal and endotracheal secretions. MMP-9 indicates matrix metalloproteinase–9; and TIMP-1, tissue inhibitor of metalloproteinase–1.

In nasal secretions, compared with baseline values, no difference was noted in active and total MMP-9 levels or the level of its natural inhibitor, TIMP-1, at hour 48 or hour 72 after treatment started in all 3 groups (Figure 2A, Figure 2B, and Figure 2C). At 48 hours after treatment began, the endotracheal aspirates showed that active MMP-9 was 0.4 log lower (99.8% CI, −1.07 to 0.28; *P* = .051) in the placebo group, 0.3 log lower (99.8% CI, −1.42 to 0.85; *P* = .39) in the standard-dose AZM group, and 0.5 log lower (99.8% CI, −1.04 to 0.14; *P* = .01) in the high-dose AZM group compared with baseline values (Figure 2D). At 72 hours after treatment began, active MMP-9 levels in endotracheal aspirates were 0.4 log lower (99.8% CI, −1.45 to 0.70; *P* = .23) in the placebo group, 0.4 log lower (99.8% CI, −1.86 to 1.05; *P* = .34) in the standard-dose AZM group, and 1.0 log lower (99.8% CI, −1.28 to −0.64; *P* < .001) in the high-dose AZM group compared with baseline values (Figure 2D).

At 48 hours after treatment began, total MMP-9 levels in endotracheal aspirates were 0.4 log lower (99.8% CI, −1.01 to 0.29; *P* = .06) in the placebo group, 0.5 log lower (99.8% CI, −1.98 to 0.89; *P* = .20) in the standard-dose AZM group, and 0.5 log lower (99.8% CI, −0.92 to 0.02; *P* = .003) in the high-dose AZM group compared with baseline values (Figure 2E). At 72 hours after treatment began, total MMP-9 in endotracheal aspirates was 0.4 log lower (99.8% CI, −1.26 to 0.52; *P* = .16) in the placebo group, 0.5 log lower (99.8% CI, −1.80 to 0.86; *P* = .23) in the standard-dose AZM group, and nearly 0.97 log lower (99.8% CI, −1.37 to −0.57; *P* < .001) in the high-dose AZM group compared with baseline values (Figure 2E). No significant difference was observed in endotracheal TIMP-1 values before and after treatment among the placebo and AZM groups (Figure 2F). Endotracheal TNF-α was 1.2 log lower (TNF-α: 99.8% CI, −1.92 to −0.47; *P* < .001) and IL-1 was 1.1 log lower (99.8% CI, −1.77 to −0.35; *P* < .001) in the high-dose AZM group 48 hours after treatment began compared with baseline (eFigures 1A and 1B in Supplement 2). Likewise, endotracheal IL-10 was 0.84 log lower (99.8% CI, −1.48 to −0.21; *P* < .001) at hour 48 and 1.2 logs lower (99.8% CI, −1.74 to −0.58; *P* < .001) 72 hours after treatment began compared with baseline in the high-dose AZM group (eFigure 1C in Supplement 2). No significant differences were observed for nasal TNF-α, IL-1, and IL-10 or for nasal and endotracheal INF-γ, IL-2, IL-6, IL-8, IL-12, and IL-13 in the placebo and both AZM groups (data not shown). Absolute concentrations of nasal active MMP-9, total MMP-9, and TIMP-1 as well as endotracheal active MMP-9, total MMP-9, TIMP-1, TNF-α, IL-1, and IL-10 are shown for pretreatment, 48 hours after treatment began, and 72 hours after treatment began in eFigure 2 in Supplement 2.

### Viral Load

At baseline, the mean (SD) RSV load measured in the endotracheal aspirate was no different among the 3 groups at 2.6 (1.0) log_10_ plaque-forming unit (PFU)/mL in the placebo group, 2.5 (1.5) log_10_ PFU/mL in the standard-dose AZM group, and 2.1 (0.8) log_10_ PFU/mL in the high-dose AZM group. On day 2 of treatment, the mean (SD) RSV load decreased to 1.2 (0.5) log_10_ PFU/mL in the placebo group, 1.2 (0.5) log_10_ PFU/mL in the standard-dose AZM group, and 0.36 (0.2) log_10_ PFU/mL in the high-dose AZM group. On day 3 of treatment, the mean (SD) RSV load was lowest in the high-dose AZM group, at 0.1 (0.1) log_10_ PFU/mL compared with 1.1 (0.4) log10 PFU/mL in the standard-dose AZM group, and 0.4 (0.3) log_10_ PFU/mL in the placebo group, but this did not reach statistical significance (*P* = .49) (eFigure 3A and 3B in Supplement 2). Nasal RSV titer correlated positively with INF-γ on day 3 (*R*^2^ = 0.47; *P* = .002). For children who were intubated, endotracheal RSV titer positively correlated with INF-γ at randomization (*R*^2^ = 0.44; *P* = .01) and, for the treatment group, at 24 hours after treatment began (*R*^2^ = 0.64; *P* = .004) and 48 hours after treatment began (*R*^2^ = 0.52; *P* = .05). No correlation was seen between viral titer and all other measured cytokines in both the nasal and endotracheal aspirate (data not shown).

### Clinical Outcomes

Table 2 summarizes the clinical outcome findings (ie, days of ventilator support, mechanical ventilation, BiPAP, HFNC, oxygenation, durations of PICU and hospital stay, and presence of multiorgan failure) for the entire study cohort as well as for the placebo and AZM treatment groups separately. Table 3 summarizes the overall treatment effect on clinical variables and by group comparisons. Median (interquartile range) length of hospital stay was lower in the high-dose group than the placebo group (8 [6-14] days vs 11 [8-20] days; mean ratio estimate, 0.57; 95 CI, 0.38-0.87; *P* = .01).

**Table 2.** Clinical Outcome Measures

Outcome	All (N = 48)	Placebo (n = 16)	AZM		*P* value for treatment effect^a^
			10 mg/kg (n = 16)	20 mg/kg (n = 16)	
Ventilator support, d^b^					
Mean (SD)	8.3 (8.7)	11.6 (13.1)	8.4 (6.1)	4.9 (2.9)	.06
Median (IQR) [range]	6 (3-11) [1-56]	7 (4-14) [2-56]	7 (3-14) [1-20]	5 (3-7) [1-11]	
Mechanical ventilation, d^c^					
Mean (SD)	4.3 (4.2)	4.8 (3.4)	5.6 (5.5)	2.6 (2.8)	.54
Median (IQR) [range]	4 (0-7) [0-16]	4 (3-8) [0-11]	5 (0-10) [0-16]	2 (0-5) [0-8]	
BiPAP, d^d^					
Mean (SD)	1.4 (7.7)	3.6 (13.2)	0.4 (1.5)	0.1 (0.3)	.02^e^
Median (IQR) [range]	0 (0-0) [0-53]	0 (0-0) [0-53]	0 (0-0) [0-6]	0 (0-0) [0-1]	
HFNC, d^f^					
Mean (SD)	2.6 (3.1)	3.3 (4.9)	2.4 (2.0)	2.3 (1.7)	.81
Median (IQR) [range]	2 (1-3) [0-18]	2 (0-3) [0-18]	2 (1-5) [0-6]	2 (1-3) [0-6]	
Oxygen, d					
Mean (SD)	9.1 (6.6)	11.0 (8.7)	9.6 (5.8)	6.6 (4.0)	.10
Median (IQR) [range]	7 (4-13) [2-31]	8 (5-16) [2-31]	8 (5-16) [2-20]	6 (4-8) [2-15]	
PICU stay, d					
Mean (SD)	7.1 (4.6)	7.7 (4.4)	8.1 (5.8)	5.4 (2.7)	.09
Median (IQR) [range]	5 (4-10) [2-19]	7 (5-10) [2-19]	6 (4-13) [2-19]	5 (4-7) [2-11]	
Hospital stay, d					
Mean (SD)	12.2 (9.1)	15.8 (13.1)	11.8 (6.5)	9.1 (4.7)	.04
Median (IQR) [range]	10 (6-16) [2-57]	11 (8-20) [3-57]	10 (7-17) [4-25]	8 (6-14) [2-16]	
Multiorgan failure					
Yes	3 (6.3)	2 (12.5)	1 (6.2)	0	
No	45 (93.7)	14 (87.5)	15 (93.8)	16 (100)	

Abbreviations: AZM, azithromycin; BiPAP, bilevel positive airway pressure; HFNC, high-flow nasal cannula; IQR, interquartile range; PICU, pediatric intensive care unit.

^a^ *P* value and estimates with 95% confidence intervals for ratio of means based on fitting a generalized linear model assuming negative binomial distribution.

^b^ Ventilator support days is the sum of mechanical ventilation, BiPAP, and HFNC days.

^c^ At randomization, there were 34 intubated patients (70.8%); 13 (81.3%) in the placebo group, 11 (68.8%) in the group receiving AZM 10 mg/kg, and 10 (62.5%) in the group receiving AZM 20 mg/kg.

^d^ At randomization, there were 2 patients (4.2%) receiving BiPAP; 1 (6.3%) in the placebo group and 1 (6.3%) in the group receiving AZM 20 mg/kg.

^e^ There was an outlier in the placebo group who received BiPAP for 53 days. This patient had congenital myopathy and was unable to wean off BiPAP and received a tracheostomy before discharge home on positive pressure ventilation. When this outlier was removed, revised analyses changed BiPAP treatment effect (*P* = .13), with pairwise comparison *P* values of .05 (AZM 20 vs AZM 10), .61 (AZM 10 vs placebo) and .13 (AZM 20 vs placebo); and ventilation support treatment effect (*P*  = .03), with pairwise comparison *P* values of .02 (AZM 20 vs AZM 10), .93 (AZM 10 vs placebo), and .02 (AZM 20 vs placebo).

^f^ At randomization, there were 12 patients (25.0%) receiving HFNC; 2 (12.5%) in the placebo group, 5 (31.3%) in the group receiving AZM 10 mg/kg, and 5 (31.3%) in the group receiving AZM 20 mg/kg.

**Table 3.** Mean Ratio Estimates for Clinical Outcomes

Outcome	AZM 20 mg/kg vs AZM 10 mg/kg		AZM 10 mg/kg vs Placebo		AZM 20 mg/kg vs Placebo	
	Mean ratio estimate (95% CI)^a^	*P* value^a^	Mean ratio estimate (95% CI)^a^	*P* value^a^	Mean ratio estimate (95% CI)^a^	*P* value^a^
Ventilator support	0.59 (0.15-2.35)	.44	0.73 (0.17-3.13)	.67	0.43 (0.21-0.86)	.02
Mechanical ventilation	0.47 (0.07-2.98)	.41	1.18 (0.20-7.10)	.85	0.55 (0.17-1.80)	.32
BiPAP	0.14 (0.02-1.18)	.07	0.12 (0.00-4.49)	.25	0.02 (0.00-0.44)	.02
HFNC	0.95 (0.30-3.04)	.93	0.73 (0.17-3.21)	.67	0.69 (0.22-2.20)	.52
Oxygen	0.69 (0.43-1.10)	.12	0.87 (0.55-1.37)	.54	0.60 (0.38-0.97)	.04
PICU stay	0.67 (0.45-1.02)	.06	1.05 (0.69-1.60)	.82	0.71 (0.49-1.02)	.07
Hospital stay	0.77 (0.53-1.13)	.18	0.74 (0.49-1.14)	.17	0.57 (0.38-0.87)	.01

Abbreviations: AZM, azithromycin; BiPAP, bilevel positive airway pressure; HFNC, high-flow nasal cannula; PICU, pediatric intensive care unit.

^a^ *P* value and estimates with 95% confidence intervals for ratio of means based on fitting a generalized linear model assuming negative binomial distribution.

## Discussion

To the best of our knowledge, this is the first randomized placebo-controlled trial of AZM at multiple doses in children with severe RSV infection and lung disease who required PICU management and positive pressure ventilation. We demonstrated preliminary efficacy of AZM, specifically at a higher dose, in the reduction of biomarkers modulated by RSV infection and in reducing duration of hospitalization in a critically ill cohort of hospitalized children.

Disease severity in RSV infection has been linked with excessive release of cytokines.^22,23,24,25,26,27^ Previous studies have shown that AZM attenuates cytokine production, which may reduce the disease severity seen in respiratory viral infections.^28^ Furthermore, preclinical studies have shown the antiviral effects of AZM.^29,30^ Given these properties, AZM has been studied for its potential use as targeted therapy for a wide spectrum of viral respiratory infections, including RSV. However, clinical trials of AZM and other macrolides in RSV infection have yielded conflicting results. In the first trial by Tahan et al,^31^ the investigators reported that treatment with clarithromycin reduced hospital length of stay and supplemental oxygen need compared with placebo. In contrast, subsequent trials using enteral AZM failed to demonstrate any clinical benefit.^32,33,34,35^ In previously reported negative trials, the study design, including patient population (non-ICU patients), macrolide dosing (1-3 doses during 1-14 days), and route of AZM administration (enteral) likely contributed to the difference seen in the findings of this trial. In a 2015 trial,^15^ treatment of RSV bronchiolitis with AZM reduced overall respiratory morbidity and, in another,^36^ decreased the progression to severe lower respiratory tract infection in preschool children.

Given the uncertainty regarding optimal dosing to achieve anti-inflammatory activity, we included a high-dose AZM group to maximize the likelihood of achieving adequate and sustained AZM levels. Others have suggested increased effectiveness of high-dose AZM in *Ureaplasma urealyticum* clearance in preterm infants.^37,38,39^ In this study, we demonstrated that high doses of AZM were safe. Although the nasal levels of MMP-9, TIMP-1, and the other RSV-related cytokines were similar among the placebo and AZM treatment groups, endotracheal active and total MMP-9, TNF-α, IL-1, and IL-10 were lower after treatment in the high-dose AZM group compared with baseline values. We also observed that RSV titer in the nasal and lung compartment decreased among all patients, and although those treated with high doses of AZM had the lowest viral burden at the end of the third dose, this finding was not significant. Although exploratory in nature, we also found that the median number of hospital days was approximately 3 days less in the high-dose group compared with the placebo group.

### Limitations

This study has limitations, including its small sample size. Although we observed decreased numbers of ventilator support and oxygen days as well as shorter duration of PICU stay in the high-dose AZM group, future studies will need to be powered to detect changes in these clinical outcome measures based on the findings of this trial. Also, it is possible that practice variation might have contributed to the clinical differences seen between the placebo vs treatment group. However, the masking of the study ensured no biases toward any particular group, and practice variation was also limited given that our unit in general follows the same respiratory weaning protocol. Furthermore, sensorineural hearing loss is a possible, although rare, adverse effect of AZM use and was not tested in our patients.

## Conclusions

We found that both doses of AZM were safe. No difference was observed in nasal MMP-9 levels across all groups. Patients who received high-dose AZM had lower endotracheal MMP-9 levels and had fewer hospitalization days. This trial provides the necessary foundation to plan a larger, multicenter randomized clinical trial that will be critical in determining the clinical effectiveness of high-dose AZM among children with severe RSV lung disease.

## Notes

Supplement 1. **Trial Procotol**

Supplement 2. **eFigure 1.** Endotracheal TNF-α, IL-1 and IL-10 Levels in the High-Dose Azithromycin Group After Treatment **eFigure 2.** Cytokine Levels Measured in the Nasal and Endotracheal Compartment Among All Groups at Baseline (Day 1), Day 2, and Day 3 **eFigure 3.** RSV Titer Measured Over Time for Patients in All 3 Groups **eTable. **Demographic and Clinical Characteristics at Baseline

Supplement 3. **Data Sharing Statement**
