# Supplementary material for: Azithromycin Treatment vs Placebo in Children With Respiratory Syncytial Virus–Induced Respiratory Failure: A Phase 2 Randomized Clinical Trial
Source: JAMA Netw Open. 2020 Apr 23;3(4):e203482. doi: 10.1001/jamanetworkopen.2020.3482 (PMC7180420; doi:10.1001/jamanetworkopen.2020.3482)
Supplement: Supplement 1. — Trial Procotol [file jamanetwopen-3-e203482-s001.pdf]

<u>Study Protocol:</u>

<u>Design</u>

The study was a double-masked, placebo-controlled phase 2 trial with 3 treatment groups (ClinicalTrials.gov NCT02707523). All subjects, their families, medical providers, and study staff were blinded to study medication allocation, with the exception of the biostatistician and the pharmacist.

<u>Recruitment and Enrollment</u>

Subject Recruitment:

16 patients per group [placebo (saline), 10mg/kg azithromycin and 20mg/kg azithromycin group; total=48] were recruited from the Pediatric Intensive Care Unit (PICU) at Children's of Alabama. All children with RSV infection and required positive pressure ventilation (invasive and non-invasive) were screened for eligibility.

Patient Compensation:

None

Race and ethnicity:

The race and ethnicity were classified based on either patient self-identification or by hospital staff during admission. Race and ethnicity were assessed in the study to provide a baseline characterization of patients cared for in this region.

<u>Eligibility/Inclusion Criteria:</u>

<u>Inclusion Criteria:</u>

1. Admission to the PICU at Children's of Alabama with a diagnosis of RSV infection and requiring positive pressure ventilation (invasive and non-invasive). Non-invasive positive pressure ventilation is defined as need for BiPAP (bilevel positive airway pressure) or high flow oxygen (namely, >1 L/kg/min of flow, with a minimum of 5 L/min flow for children <5 kg).

2. Enrollment into the study within 48 hours of ICU admission and placement on positive pressure ventilation

3. Age 0-16 years

<u>Exclusion Criteria:</u>

1. AZM use within 7 days of PICU admission

2. Contraindication to AZM use

- Patients with electrocardiogram QT interval corrected for heart rate (Qtc) ≥ 450 ms
- Patients with significant hepatic impairment (direct bilirubin >1.5 mg/dL)
- Known hypersensitivity to azithromycin, erythromycin, any macrolide, or ketolide drug
- Cardiac arrhythmia

3. History of pyloric stenosis

4. Patients requiring chemotherapy or on chronic steroids for malignancy or post bone marrow transplantation

5. Positive pressure ventilation greater than 48 hours prior to PICU admission

<u>Informed Consent:</u>

Consent was obtained in a quiet setting prior to the initiation of the study. The legal guardian was given opportunity to delay consent until he/she has taken adequate time to read and understand the written consent and discuss the study with others. Subjects were not consented in writing

until they were able to demonstrate adequate understanding of all aspects of the study and consent process. A copy of the consent form was given to the patient/legal guardian. The signed consent form remained in the patient's study files at the clinical center.

Randomization:

All enrolled subjects were randomized according to a permuted-block design to receive either placebo (saline) or AZM (Fresenius Kabi, USA) at 10mg/kg/day (referred to as "standard dose") or 20mg/kg/day (referred to as 'high dose") intravenously (IV) every 24 hours for 3 days. The randomization schedule was created by the study biostatistician and provided to the study pharmacist for implementation. The FDA required the study enrollment to be done in two stages, Stage 1: First 9 subjects (older than 6 months), and Stage 2: Remaining 39 subjects (of all ages) if no safety issues were found. The first stage enrolled 9 subjects equally to the 3 treatment groups. For this, the random assignment of treatment was determined by random permutation. For the second stage, we randomly assigned the 3 treatments equally to the 39 subjects using block randomization of size 3 or 6. In addition, to maintain blinding to the treatment assignment, placebo was randomly assigned to either 10 mg/kg or 20 mg/kg dose. Dr. Aban generated the randomization schedule as described above. Dr. Aban provided the randomization schedule (with randomization IDs) in an excel file to the study pharmacist in charge of dispensing the appropriate treatment.

Duration of Study:

The trial began in February of 2016, and due to the age restriction placed by the FDA, the first 9 subjects enrolled were older than 6 months of age. Once this number was attained, and no safety

concerns were evident as determined by the DSMB, infants younger than 6 months of age were eligible for enrollment in the study. The study spanned three and a half RSV seasons, with the last patient enrolled in February of 2019. The trial ended once 48 subjects were enrolled into the study. All subjects completed the study. No long term follow up was provided.

Primary and Secondary Outcomes

The primary outcomes of this trial were to ascertain 1. The safety and AZM pharmacokinetic and 2. If treatment with AZM resulted in decreased nasal MMP-9 levels. Notably, when the study protocol was expanded to include non-ventilated patients, the primary outcome also changed from endotracheal MMP-9 to nasal MMP-9 levels. The secondary outcomes were to determine the *in vivo* efficacy of AZM administration on improving clinical outcome measures, specifically duration of ventilatory support and oxygenation, as well as PICU and hospital length of stay. The CONSORT guideline was used for the reporting of this trial.

Visit Schedule and Study Procedures

Overview:

If subjects met the inclusion criteria and have no contraindication to study participation, they were be enrolled into the study within 48 hours of positive pressure ventilation. Subjects were randomized via a permuted block design to receive either placebo (normal saline), 10mg/kg of IV azithromycin or 20 mg/kg of IV azithromycin, daily for 3 days. Prior to drug administration, assessment was performed to ensure that subjects do not have contraindications of AZM administration or development of any adverse events. Nasal and endotracheal aspirate (if

intubated) were obtained daily for the first 3 days (first sample obtained before drug administration).

Prior to drug administration, baseline labs (CBC with differential, liver and renal function, electrolytes) was reviewed. If the patient had evidence of hypokalemia, hypocalcemia or hypomagnesemia, the electrolyte abnormalities were corrected prior to drug administration. EKG was also reviewed, and if there was evidence of prolonged QT, the subject was excluded from the study. Vital signs (heart rate, blood pressure, temperature, saturations), ventilator or BiPAP or HFNC settings, presence of multi-organ failure were recorded. All these parameters were assessed prior to drug administration. Vital signs (heart rate, blood pressure, temperature, saturations) were also assessed immediately before the drug infusion, during, and 2 and 4 hours post completion of infusion.

During the hospitalization, all patients were treated according to the American Academy of Pediatrics guidelines for the management of bronchiolitis, namely supportive care. All decisions regarding medical treatment during hospitalization were made by the patient's primary care team.

Data Collection:

Nasal, endotracheal aspirates (if intubated) and serum samples were obtained once daily for the first 3 days. The collection window had at least 24 hours between each sample collection. If patients were extubated within the first 3 days of study window, only nasal samples will be collected. Clinical data was collected each day until the subject was discharged from the ICU.

An overview of clinical and biologic data is outlined in this table.

	Day 1	Day 2	Day 3	Day of PICU discharge	Day of hospital discharge
AZM level (serum, nasal, lung)	X	X	X		
RSV titer (nasal and lung)	X	X	X		
Active MMP-9, Total MMP-9, TIMP (nasal and lung)	X	X	X		
Other RSV cytokine activity (nasal and lung)	X	X	X		
Continous vital signs monitoring[1]	X	X	X		
Baseline and continous lab review[2]	X	X	X		
Electrocardiogram	X	X	X		
Study drug administration[3]	X	X	X		
Duration of positive pressure ventilation (MV, BiPAP, HFNC)	X	X	X	X	X
Duration of oxygenation	X	X	X	X	X
Duration of PICU stay				X	
Duration of hospital stay					X

[1]All assessments were done prior to dosing, during dosing until completion of infusion, immediately at the end of infusion, 2 and 4 hours post completion of infusion.

[2]Laboratories that were collected: CBC with differential, electrolytes, liver and renal function; record if done for clinical care.

[3]Study drug administration took place on 3 consecutive days (1 dose per day) after consenting. Study drug at 2 mg/ml was administered over 60 minutes.

Withdrawal of Participant Consent and Discontinuation of Study Drug:

Study participation may be discontinued for any of the following reasons:

a. Subject decision to withdraw consent for study

b. Evidence of allergy to administered products

c. Intolerable adverse event as judged by study investigator

Safety Data

Adverse Events:

a. Adverse event: Any untoward medical occurrence in a patient or clinical investigation in which a subject is given a pharmaceutical product; does not necessarily have a causal relationship with such treatment, or

b. Any unfavorable and unintended sign (including abnormal laboratory findings), symptom, or disease temporally associated with the use of a medicinal (investigational) product; not necessarily related to the product.

c. Change-from-baseline

AEs can take 2 forms:

i. Appearance of a new symptom or sign, or

ii. Increased severity or frequency of an existing symptom or sign

d. Severity assessment: Severity refers to the intensity of the event and is generally indicated as mild (Grade I), moderate (Grade II), severe (Grade III), or life-threatening (Grade IV). Grading definitions are:

i. Mild (Grade I): mild transient symptoms, only requiring monitoring or symptomatic treatment, and clinically significant

ii. Moderate (Grade II): Moderate illness or condition which requires new or significantly altered specific therapy

iii. Severe (Grade III): Severe illness or condition: unresponsive to medical therapy.

iv. Life-threatening: Life-threatening illness or condition. Complicated by acute, life-threatening metabolic or cardiovascular complications (such as circulatory failure, hemorrhage, sepsis); life-threatening physiological consequences; or need for intensive care or emergent invasive procedure (e.g. requires major surgery).

e. Causality refers to the likelihood and extent that the investigational agent being studied contributed to the development of an AE. In making determination, consider temporal relationship to drug exposure, other possible exposures as etiology, known effects of the investigational agent, anticipated (expected) side effects derived from preclinical studies, and what is known about similar drugs (See drug insert).

Relatedness of AEs to an Intervention (Agent)

Definite (must have all 4)

• Has a reasonable temporal relationship to the intervention

• Could not have readily been produced by the subject's clinical state or have been due to environmental or other interventions

• Follows a known pattern of response to intervention

• Disappears or decreases with reduction in dose or cessation of intervention and recurs with re-exposure

Probable (must have 3)

• Has a reasonable temporal relationship to the intervention

• Could not have readily been produced by the subject's clinical state or have been due to environmental or other interventions

• Follows a known pattern of response to intervention

• Disappears or decreases with reduction in dose or cessation of intervention

Possible (must have 2)

• Has a reasonable temporal relationship to the intervention

• Could not have readily been produced by the subject's clinical state

• Could not readily have been due to environmental or other interventions

• Follows a known pattern of response to intervention

Unlikely (must have 2)

• Does not have a temporal relationship to the intervention

• Could readily have been produced by the subject's clinical state

• Could have been due to environmental or other interventions

• Does not follow a known pattern of response to intervention

• Does not reappear or worsen with reintroduction of intervention

f. Duration of event: Onset of signs and symptoms as well as resolution of the event should be recorded, typically defined as the point when all signs and symptoms have subsided. Some events continue or change in severity over time. Capturing this information is also important. Changes in severity such as when an event improves from severe to mild should be recorded as separate events, with separate intensities and durations collected.

g. Adverse reaction: Defined as an adverse event caused by a drug. Therefore, adverse reactions are a subset of all suspected adverse reactions for which there are reasons to conclude that the drug caused the event.

h. Suspected adverse reaction (21 CFR 312.32(a)): Suspected adverse reaction means any adverse event for which there is a reasonable possibility that the drug caused the event. Reasonable possibility means there is evidence to suggest a causal relationship between the drug and the adverse event. A suspected adverse reaction implies a lesser degree of certainty about causality than adverse reaction, which means any adverse event caused by a drug.

i. Unexpected (21 CFR 312.32(a)): An adverse event or suspected adverse reaction is considered “unexpected” if it is not listed in the investigator brochure (or drug insert) or is not listed at the specificity or severity that has been observed; or, is not consistent with the risk information described in the general investigational plan or elsewhere in the protocol. “Unexpected” also refers to adverse events or suspected adverse reactions that are mentioned in the investigator brochure as occurring with a class of drugs or as anticipated from the pharmacologic properties of the drug, but are not specifically mentioned as occurring with the particular drug under investigation (e.g. adverse events associated with other macrolides, but not specifically AZM such as pyloric stenosis). In addition, adverse events that would be anticipated to occur as part of

the disease process are considered ‘unexpected’ for the purposes of reporting because they would not be listed in the investigator brochure.

j. Serious (21 CFR 312.32(a)): An adverse event or suspected adverse reaction is considered ‘serious’ if, in the view of either the investigator or sponsor, it results in any of the following outcomes: death, a life-threatening adverse event, inpatient hospitalization, or prolongation of existing hospitalization, a persistent or significant incapacity, or important medical event that may, based on appropriate medical judgment, jeopardize the patient or subject and may require medical or surgical intervention to prevent one of the other outcomes listed in the definition.

k. Life-threatening (21 CFR 312.32(a)): An AE or suspected adverse reaction is considered ‘life-threatening if, in the view of the investigator or sponsor, its occurrence places the patient or subject at immediate risk of death.

<u>SAEs</u>

Serious (21 CFR 312.32(a)): An adverse event or suspected adverse reaction is considered ‘serious’ if, in the view of either the investigator or sponsor, it results in any of the following outcomes: death, a life-threatening adverse event, inpatient hospitalization, or prolongation of existing hospitalization, a persistent or significant incapacity, or important medical event that may, based on appropriate medical judgment, jeopardize the patient or subject and may require medical or surgical intervention to prevent one of the other outcomes listed in the definition.

Life-threatening (21 CFR 312.32(a)): An AE or suspected adverse reaction is considered ‘life-threatening if, in the view of the investigator or sponsor, its occurrence places the patient or subject at immediate risk of death.

Safety Monitoring: An independent Data and Safety Monitoring Board (DSMB) was appointed. Members included two pediatric intensivist, a biostatistician, a clinical trials epidemiologist, and an infectious disease specialist. The DSMB 1) reviewed and analyzed the progress of the study, 2) approved amendments to the trial protocol, 3) monitored the safety of the study treatments and 4) reviewed data quality. Monitoring for serious or unexpected adverse events (SAEs) was performed throughout the ICU period. The DSMB met before subject recruitment begun and subsequently meet every 4-6 months in person or by teleconference to review the study progress and AE and SAE reports.

Data Processing and Management

All participant data were obtained and recorded in individual files that was stored in a secured file cabinet within a locked office. Upon study entry, all participants were given a unique identifier to be used through the entirety of the study and this too was maintained in a locked file cabinet in secured office. Following each study visit, the data obtained was entered into a database using the study participants' unique identifiers. All other personal data were omitted from electronic records. At the close of the study, any missing data was verified with chart review. Only authorized study personnel have access to patient data.

Data Analysis

The primary outcome measured was MMP-9 activity. Based on our preliminary data, we expect that n=16 per group would be sufficient to detect a 20% and 25% decrease in MMP-9 activity in the treated group (10mg/kg and 20mg/kg; respectively) relative to placebo with 80% power.

Inmaculada Aban, Ph.D (Professor, UAB School of Public Health) is the biostatistician for this clinical trial.

Nasal and Endotracheal Aspirate Collection and Processing

Nasal samples were collected using a nasal swab inserted through the nares parallel to the palate (not upwards) until resistance was encountered or the distance inserted was equivalent to that from the nostril to the ear of the patient indicating contact with the nasopharynx. After gentle rubbing and rolling of the swab, it was left in place for several seconds to absorb secretions prior to removal. The swab was then immediately placed into a sterile vial containing 3 ml of viral transport media (Quidel, San Diego, CA), and kept at 4°C for further analysis.

Endotracheal aspirates were collected via endotracheal tube suctioning using an 8 Fr suction cannula (CareFusion, San Diego, CA, USA) and centrifuged at 500xg for 10 min to separate supernatant from cells and mucus (pellet) . Separate aliquots of supernatant were subsequently saved at 4°C for further analysis.

Assay of AZM, Urea and Pharmacokinetic Analysis

AZM and urea levels in tracheal aspirates and plasma samples were measured using liquid chromatography-tandem mass spectrometric assays. For quantitation of AZM, all samples were treated with 200 µL of acetonitrile containing terfenadine (5 ng/mL) as an internal standard. Samples were then vortexed briefly and centrifuged at 20,000xg for 5 minutes. The supernatant was transferred to autosampler vials for analysis. Detection was performed using an Applied BioSystems 4000 QTRAP (Applied Biosystems, Foster City, CA) triple quadrupole mass

spectrometer operated in the positive ion mode. Separation of AZM and the internal standard from the biological matrix was achieved using a Phenomenex Luna C18, 100 X 2 mm 5 µm particle column. The mobile phase was delivered at a flow rate of 400 µL/min using a gradient elution profile consisting of DI water with 0.1% formic acid (A) and acetonitrile with 0.1% formic acid (B). The AZM and internal standard were detected using multiple reaction monitoring (MRM) for the following transitions: AZM (m/z 749.6→591.5), and Terfenadine (m/z 472.4→436.3).

Samples (20 µL) for urea analysis were prepared by diluting each sample matrix with 955 µL of 90:10 acetonitrile:water followed by the addition of 25 µL of internal standard (10 µg/mL 4-aminobenzoic acid). The samples were vortexed, centrifuged and the resulting supernatant was transferred to autosampler vials and analyzed by LC/MS/MS. Separation of urea and the internal standard from the biological matrix was achieved using a Waters Spherisorb Silica, 150 X 3.2 mm 5 µm particle column (Waters Corporation, Milford, MA). The mobile phase was delivered at a flow rate of 400 µL/min using a gradient elution profile consisting of DI water with 0.1% formic acid (A) and acetonitrile with 0.1% formic acid (B). The urea and internal standard were detected using multiple reaction monitoring (MRM) for the following the following transitions: Urea (m/z 61.1→44.1), and 4-aminobenzoic acid (m/z 138.1→120.1).

Dilution estimations of recovered samples from endotracheal compartment were calculated using the urea dilution method. The following equation was used to calculate the corrected drug level in these samples:

$$AZM_{corrected} = \frac{Urea_{plasma}}{Urea_{matrix}} \; X \; AZM_{measured}$$

Where $Urea_{matrix}$ was the amount of urea in the endotracheal samples, $Urea_{plasma}$ was the amount of urea in the plasma of the corresponding patient, $AZM_{measured}$ was the measured amount of AZM in the matrix being assayed.

Peak plasma levels (C_{max}) and time to C_{max} (T_{max}) of AZM in all matrices were determined by visual evaluation of the concentration versus time profile. The AUC (area under the curve) values from 0 to 144 hours for each matrix was calculated using the trapezoidal rule. The half-life ($t_{1/2}$) of AZM in plasma and endotracheal samples were calculated as ln(2)/ke, where ke is the terminal elimination rate constant estimated by linear regression analysis of the terminal portion of the concentration-time profile.

Penetration ratios for AZN in samples from the endotracheal compartment were calculated as $AUC_{endotracheal}/AUC_{plasma.}$

Biologic Outcome Measurement

Measurement of active MMP-9, total MMP-9 and Tissue Inhibitor of Metalloproteinase (TIMP)-1 was done using an established fluorometric assay (#F9M00 and #DTM100, respectively; R&D Systems, Calbiochem, Billerica, MA, USA). Interleukin (IL)-1, IL-2, IL-4, IL-6, IL-8, IL-10, IL-12, IL-13, tumor necrosis factor (TNF)-α, and interferon (IFN)-γ were analyzed via electrochemiluminescence using multi-array plates on a Meso Scale Discovery (MSD) Sector Imager 2400 (MSD, Gaithersburg, MD) using the MSD V-PLEX Cytokine Panel 1 Human Kit

(No. K15049D-1, Rockville, Maryland, USA). All samples were processed as indicated by the manufacturer's protocol.

In brief, RSV loads were measured by reverse-transcription (RT) quantitative PCR using known concentrations of RSV to derive a standard curve. Standards and negative controls were included and tested with each PCR assay. RSV quantification was reported as log10 copies/mL.

Clinical Data Assessment

The clinical data collected included duration of respiratory support, focusing on days of mechanical ventilation, non-invasive positive pressure ventilation via BiPAP or high flow nasal cannula, and total duration of supplemental oxygen. Other clinical data assessed included length of PICU and hospital stay. Co-morbidities such as history of prematurity, apnea, seizure disorder, chromosomal abnormalities, and the presence of multi-organ failure were identified.

Sample Size Determination and Statistical Analysis

This study was designed to have a total sample size of 48 participants divided into three groups, with 16 per group to detect a 25% and 37.5% decrease in lung MMP-9 activity in the treated groups (10mg/kg and 20mg/kg, respectively) relative to placebo with 80% power using analysis of variance. The assumptions were based on MMP-9 levels found in endotracheal aspirate of children with RSV-induced respiratory failure (14).

Means, standard deviations, medians, quartiles and range (minimum, maximum) described continuous variables and counts and percentages for categorical variables. To compare

baseline characteristics among the treatment groups, we used Kruskal-Wallis test for age, height and weight due to evidence of non-normality of the distribution of the values. We used either Chi-Square or Fisher's exact test for categorical variables depending on expected cell counts.

We analyzed inflammatory markers by first performing $\log_{10}$ transformation to address the skewness of the distribution and extreme outliers. We then calculated the difference at each time point relative to baseline to obtain the change (i.e. pre and post treatment) outcome and fitted a generalized linear mixed model (GLMM) with random intercepts and assumed unequal variances across treatment groups with change from baseline as the outcome. The model included treatment, day and treatment by day interaction. Point and confidence interval estimates of the mean change outcome at each time point and treatment group combination were obtained. If a confidence interval for the mean change does not include 0, this is evidence of an effect associated with that particular treatment as it is either a significant increase (or decrease) in the inflammatory marker relative to baseline. The Bonferroni approach was used to adjust for multiple testing and simultaneous confidence intervals. Thus, significance was determined using a cut-off of 0.002 and a 99.8% level was used to construct confidence intervals for each for each treatment at each time point. Note that the sample size justification was based on the assumption that the MMP-9 levels are normally distributed with common standard deviation. However, in the data for this trial, we observed evidence of non-normality and non-constant variance across the treatment groups. Thus, GLMM was deemed more appropriate than ANOVA. Furthermore, fitting GLMM allows us to combine unbalanced data from different time points to help gain more power in evaluating treatment effect.

We used a generalized linear model assuming a negative binomial distribution for clinical

outcomes (measured in number of days), using log link to address possible overdispersion due to some extreme observations. Furthermore, we fitted a model that allowed the variance to differ by treatment group. Spearman Correlation was used to examine associations. For this set of analyses, the standard 5% level of significance and 95% confidence interval was used as these are all exploratory outcomes. All analyses were done using SAS version 9.4.

IRB and IND

The study protocol was approved by the University of Alabama at Birmingham Institutional Review Board. An Investigational New Drug (IND) was obtained from the U.S Food and Drug Administration (FDA) for the use of AZM in this trial (IND 127632). An age restriction was placed by the FDA at the onset of enrollment for the first 9 patients whereby only infants older than 6 months of age could be screened and potentially enrolled in the study.
