# Supplementary material for: Azithromycin Treatment vs Placebo in Children With Respiratory Syncytial Virus–Induced Respiratory Failure: A Phase 2 Randomized Clinical Trial
Source: JAMA Netw Open. 2020 Apr 23;3(4):e203482. doi: 10.1001/jamanetworkopen.2020.3482 (PMC7180420; doi:10.1001/jamanetworkopen.2020.3482)
Supplement: Supplement 2. — eFigure 1. Endotracheal TNF-α, IL-1 and IL-10 Levels in the High-Dose Azithromycin Group After Treatment eFigure 2. Cytokine Levels Measured in the Nasal and Endotracheal Compartment Among All Groups at Baseline (Day 1), Day 2, and Day 3 eFigure 3. RSV Titer Measured Over Time for Patients in All 3 Groups eTable. Demographic and Clinical Characteristics at Baseline [file jamanetwopen-3-e203482-s002.pdf]

Supplementary Online Content

Kong M, Zhang WW, Sewell K, et al. Azithromycin treatment vs placebo in children with respiratory syncytial virus–induced respiratory failure: a phase 2 randomized clinical trial. *JAMA Netw Open*. 2020;3(4):e203482. doi:10.1001/jamanetworkopen.2020.3482

eFigure 1. Endotracheal TNF-α, IL-1 and IL-10 Levels in the High-Dose Azithromycin Group After Treatment
eFigure 2. Cytokine Levels Measured in the Nasal and Endotracheal Compartment Among All Groups at Baseline (Day 1), Day 2, and Day 3
eFigure 3. RSV Titer Measured Over Time for Patients in All 3 Groups
eTable. Demographic and Clinical Characteristics at Baseline

This supplementary material has been provided by the authors to give readers additional information about their work.

© 2020 Kong M et al. *JAMA Network Open.*

eFigure 1. Endotracheal TNF-α, IL-1 and IL-10 Levels in the High-Dose Azithromycin Group After Treatment

eFigure 1a: Decreased endotracheal TNF-α in the high dose AZM group post treatment

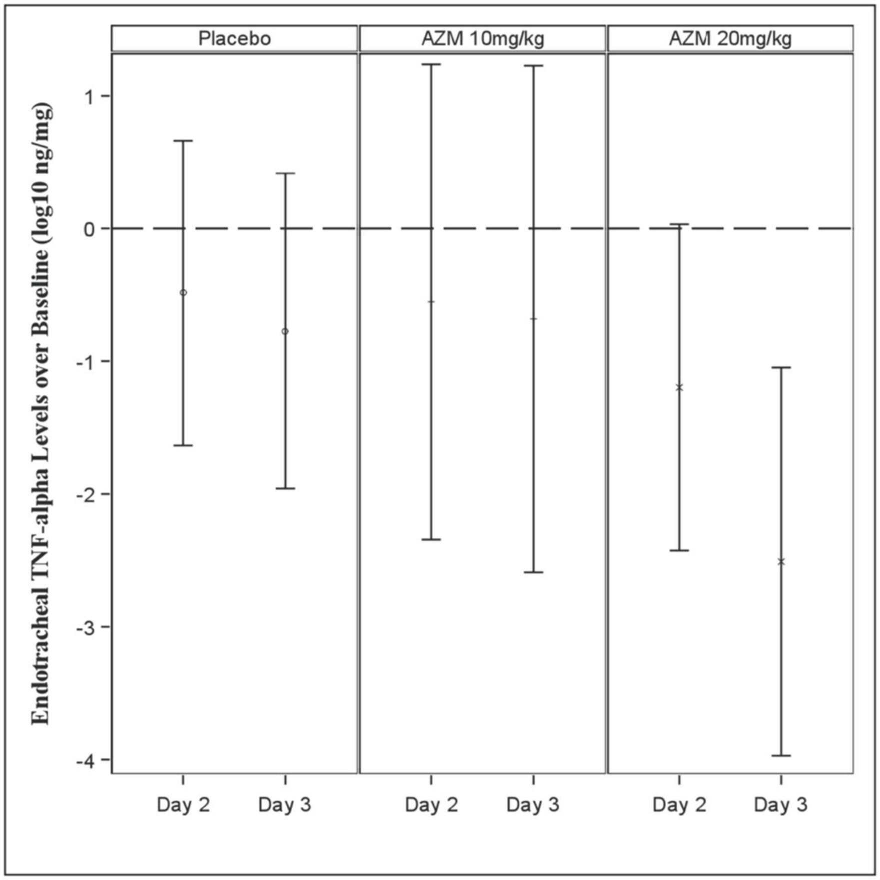

TNF-α in the endotracheal aspirate was approximately 1.2 log lower in the high dose AZM group on day 2 (99.8% CI: -1.92,0.47; p<0.001) and 2.5 logs lower on day 3 (99.8% CI: -5.76,0.74; p=0.01) relative to baseline. In the standard AZM group, no difference was observed on day 2 (99.8% CI: -2.13,1.03; p=0.23) and day 3 (99.8% CI: -2.88,1.51; p=0.28) relative to baseline. In the placebo group, TNF-α was not different on day 2 (99.8% CI: -1.45,0.48; p=0.09) and 0.77 log lower on day 3 (99.8% CI: -1.96,0.42; p=0.03) relative to baseline.

© 2020 Kong M et al. *JAMA Network Open.*

eFigure 1b: Decreased endotracheal IL-1 in the high dose AZM group post treatment

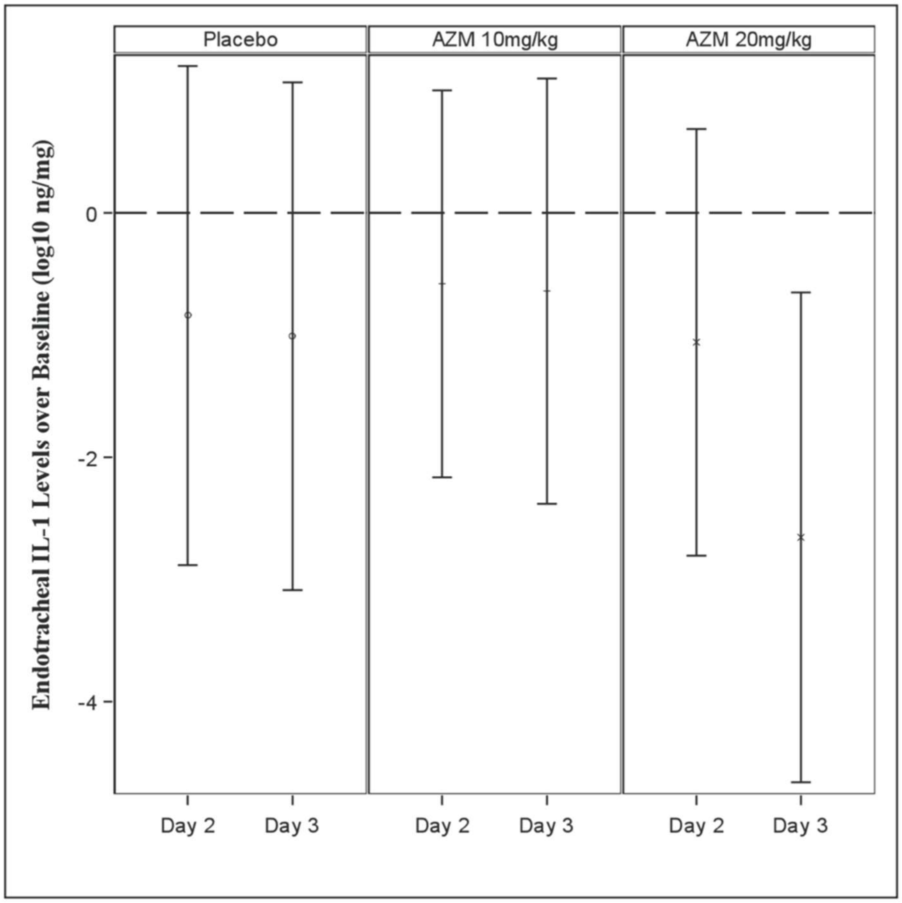

IL-1 in the endotracheal aspirate was approximately a log lower in the high dose AZM group at day 2 post treatment (99.8% CI: -1.77,-0.35; p<0.001) and 2.7 logs lower on day 3 (99.8% CI: -7.14,1.83; p=0.05) relative to baseline. No difference was observed between day 2 (99.8% CI: -1.94, 0.78; p=0.15) and day 3 (99.8% CI: -2.41, 1.14; p=0.22) endotracheal IL-1 levels compared to baseline for the standard AZM group. Similarly, in the placebo group, no difference was observed on day 2 (99.8% CI: -2.85,1.18; p=0.16) and day 3 (99.8% CI: -3.07,1.05; p=0.10) relative to baseline.

© 2020 Kong M et al. *JAMA Network Open.*

eFigure 1c: Decreased endotracheal IL-10 in the high dose AZM group post treatment

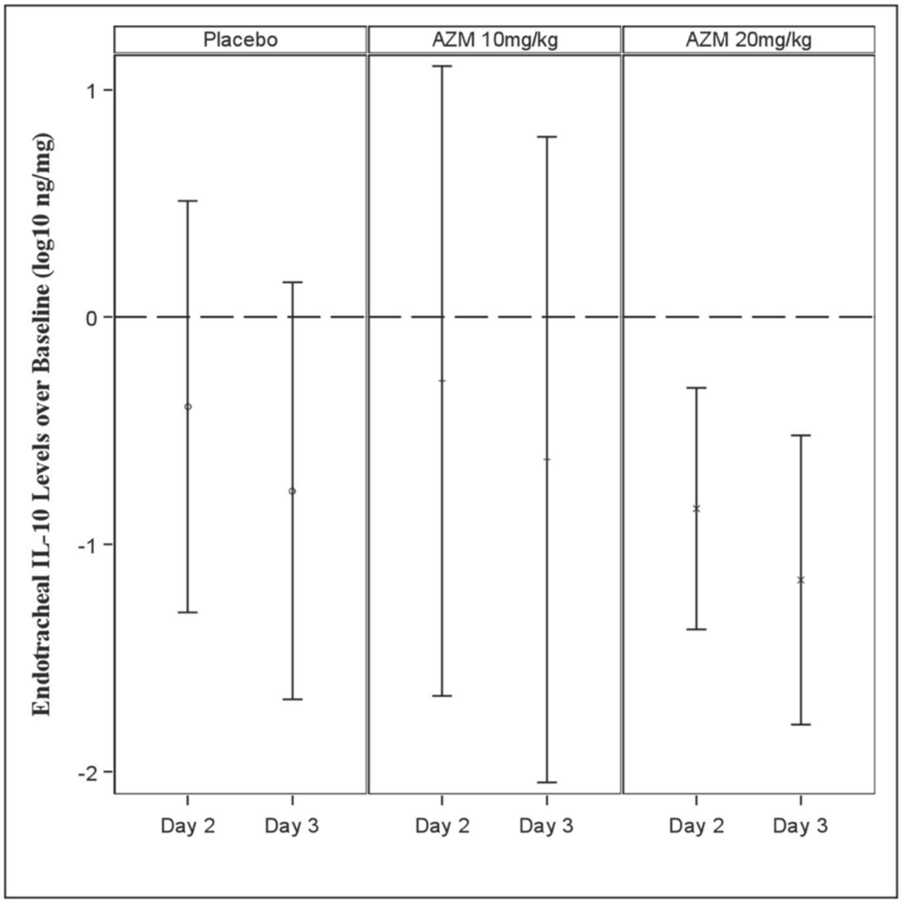

IL-10 in the endotracheal aspirate was 0.84 (99.8% CI: -1.48, -0.21; p=0.001) and 1.16 (99.8% CI: -1.74,-0.58; p<0.001) log units lower post treatment on days 2 and 3, respectively, compared to baseline after treatment in the high dose AZM group. No difference was observed between day 2 in the placebo group (99.8% CI: -1.25, 0.46; p=0.12) and the standard dose AZM group (99.8% CI: -1.76, 1.2; p=0.51) compared to baseline. On day 3, IL-10 was 0.76 log lower (99.8% CI: -1.77, 0.24; p=0.01) in the placebo group, and no different in the standard dose group (99.8% CI: -2.21, 0.96; p=0.18), respectively.

© 2020 Kong M et al. *JAMA Network Open.*

eFigure 2. Cytokine Levels Measured in the Nasal and Endotracheal Compartment Among All Groups at Baseline (Day 1), Day 2, and Day 3

eFigure 2a: Nasal Active MMP-9 Levels

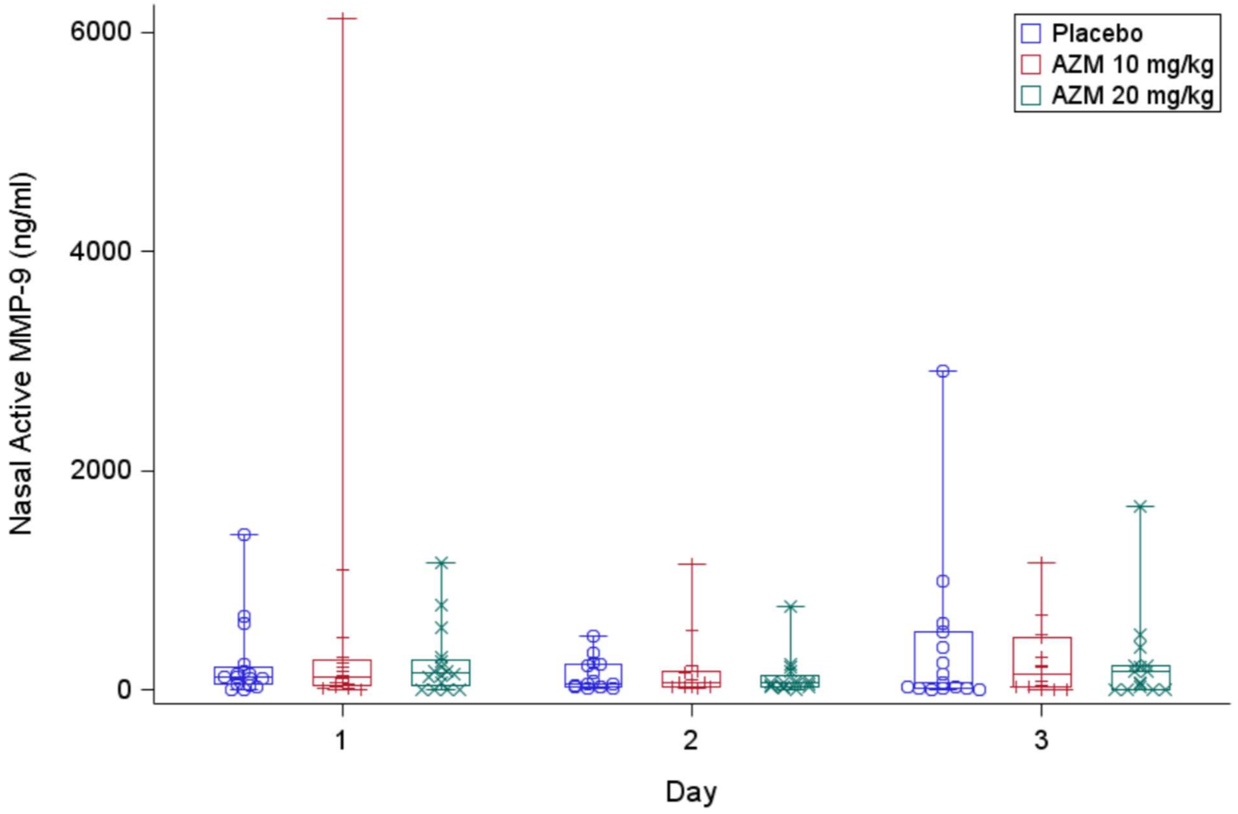

At baseline, the median (intra-quartile range, IQR) of nasal active MMP-9 in the placebo group was 117 (154) ng/ml vs. 120 (234) ng/ml in the standard AZM group, and 156 (243) ng/ml in the high dose AZM group. On day 2, the median (IQR) of nasal active MMP-9 was 51 (206) ng/ml in the placebo group vs. 70 (136) ng/ml in the standard AZM group, and 60 (97) ng/ml in the high dose AZM group. On day 3, the median (IQR) of nasal active MMP-9 was 64 (513) ng/ml in the placebo group vs. 142 (456) ng/ml in the standard AZM group, and 166 (215) ng/ml in the high dose AZM group. Upper and lower bars are maximum and minimum observations.

© 2020 Kong M et al. *JAMA Network Open.*

eFigure 2b: Nasal Total MMP-9 Levels

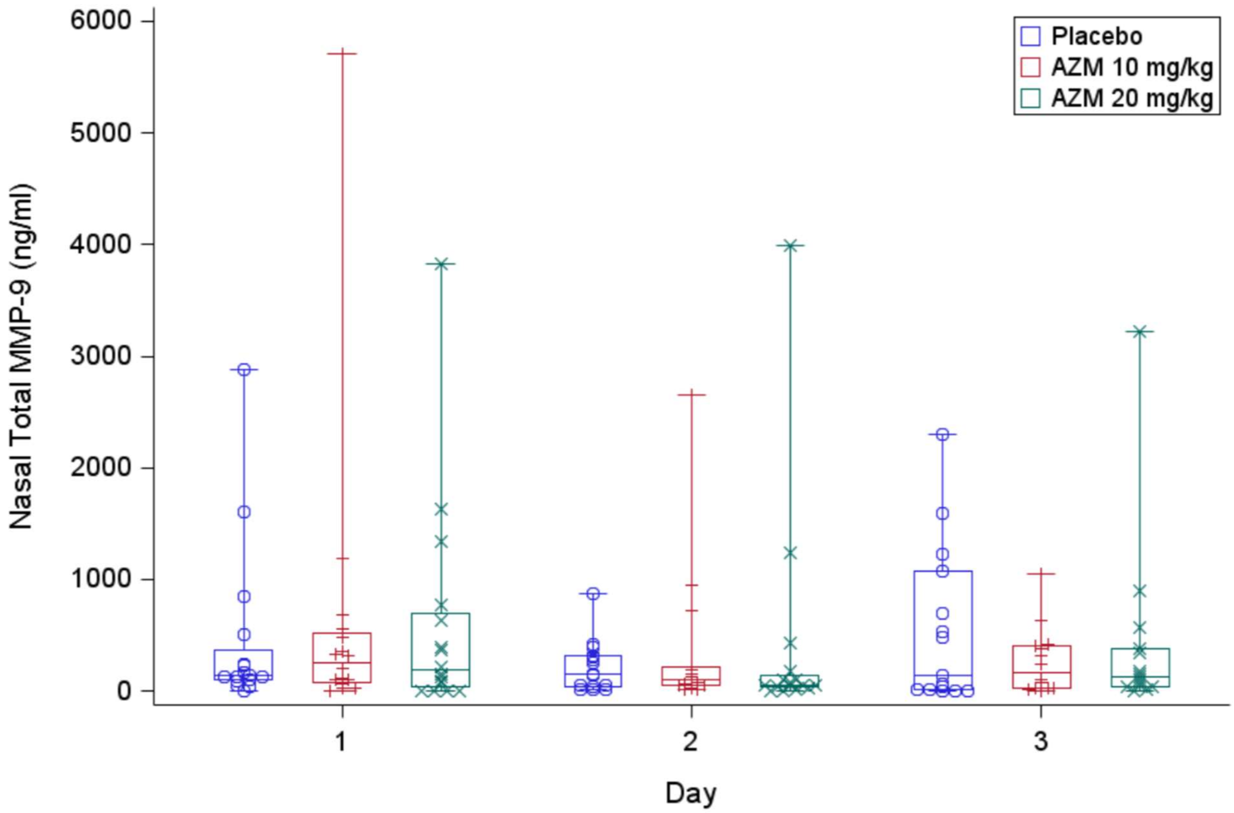

At baseline, the median (IQR) of nasal total MMP-9 in the placebo group was 135 (268) ng/ml vs. 259 (434) ng/ml in the standard AZM group, and 189 (661) ng/ml in the high dose AZM group. On day 2, the median (IQR) of nasal total MMP-9 was 147 (274) ng/ml in the placebo group vs. 103 (163) ng/ml in the standard AZM group, and 56 (106) ng/ml in the high dose AZM group. On day 3, the median (IQR) of nasal total MMP-9 was 135 (1064) ng/ml in the placebo group vs. 169 (375) ng/ml in the standard AZM group, and 122 (329) ng/ml in the high dose AZM group. Upper and lower bars are maximum and minimum observations.

© 2020 Kong M et al. *JAMA Network Open.*

eFigure 2c: Nasal TIMP-1 Levels

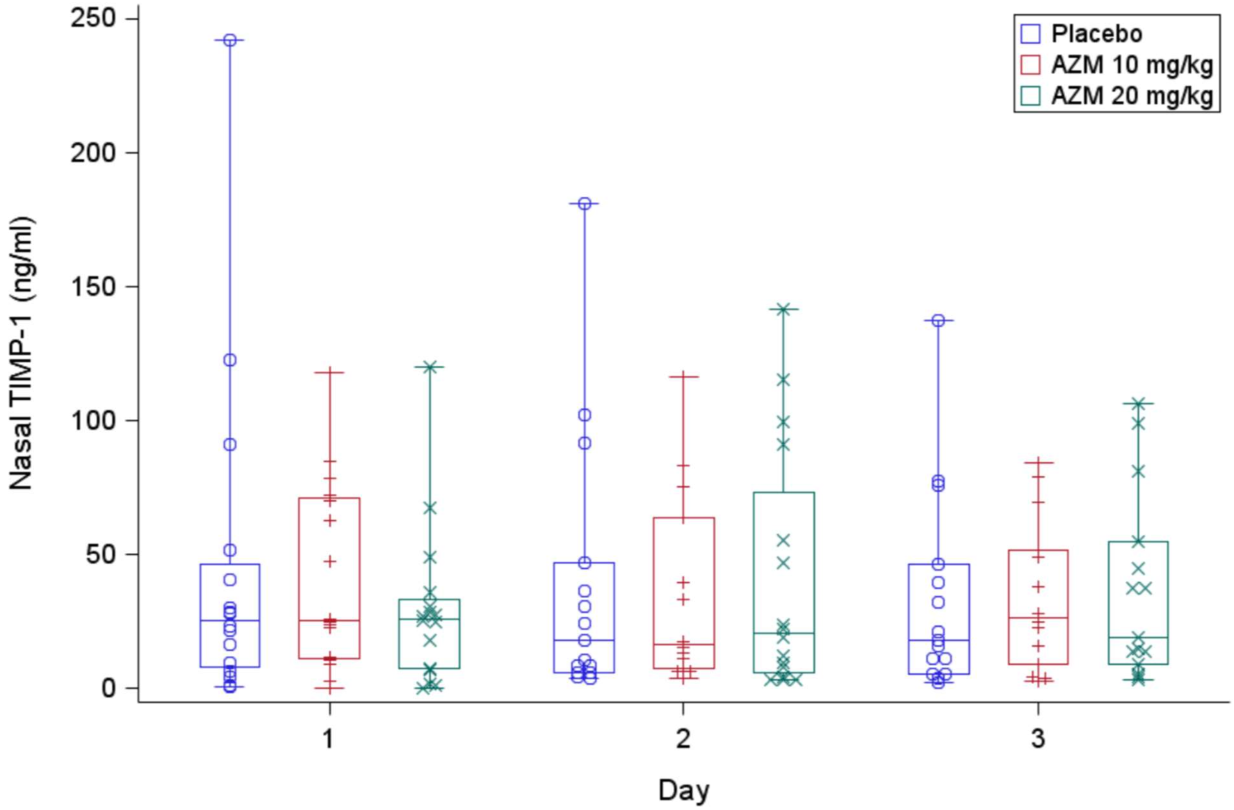

At baseline, the median (IQR) of nasal total TIMP-1 in the placebo group was 26 (38) ng/ml vs. 26 (60) ng/ml in the standard AZM group, and 26 (25) ng/ml in the high dose AZM group. On day 2, the median (IQR) of nasal total TIMP-1 was 18 (41) ng/ml in the placebo group vs. 16 (56) ng/ml in the standard AZM group, and 21(67) ng/ml in the high dose AZM group. On day 3, the median (IQR) of nasal total TIMP-1 was 18 (40) ng/ml in the placebo group vs. 27 (42) ng/ml in the standard AZM group, and 19 (45) ng/ml in the high dose AZM group. Upper and lower bars are maximum and minimum observations.

© 2020 Kong M et al. *JAMA Network Open.*

eFigure 2d: Endotracheal Active MMP-9 Levels

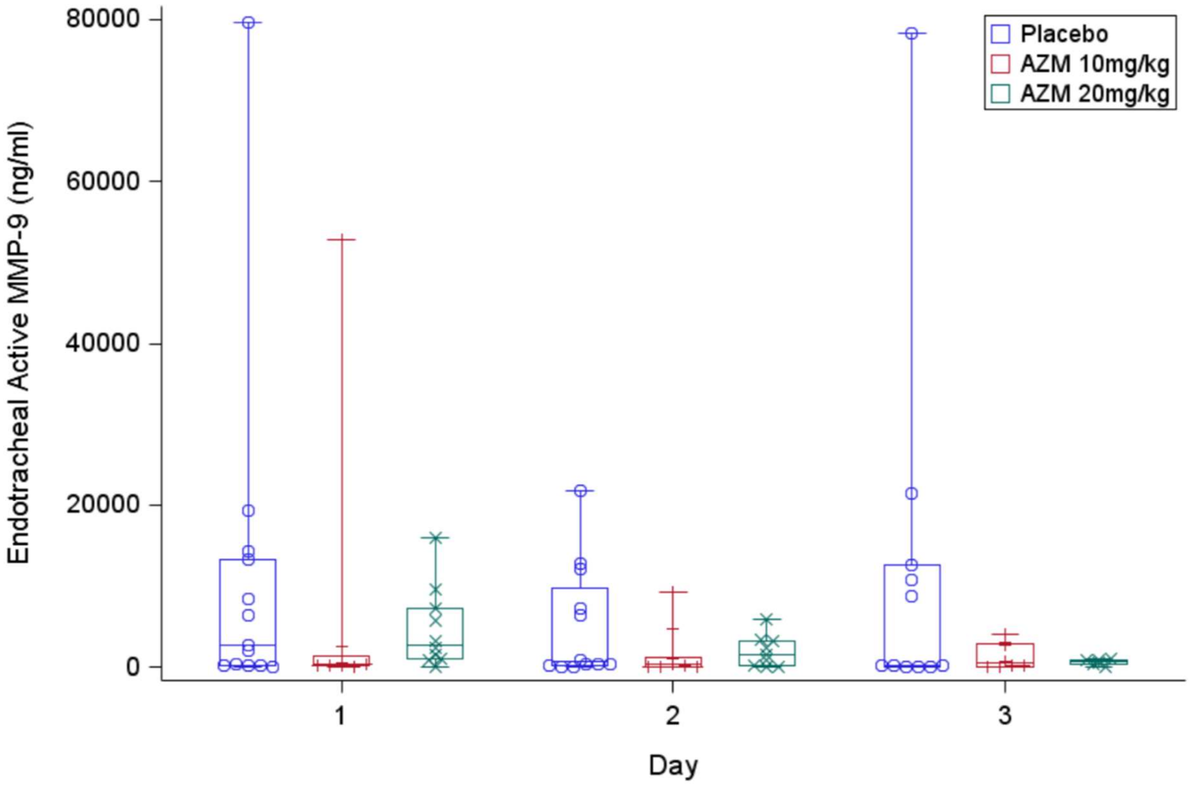

At baseline, the median (IQR) of endotracheal active MMP-9 in the placebo group was 2686 (13048) ng/ml vs. 394 (1170) ng/ml in the standard AZM group, and 2764 (6185) ng/ml in the high dose AZM group. On day 2, the median (IQR) of endotracheal active MMP-9 was 625 (9428) ng/ml in the placebo group vs. 336 (1118) ng/ml in the standard AZM group, and 1521 (3048) ng/ml in the high dose AZM group. On day 3, the median (IQR) of endotracheal active MMP-9 was 253 (12474) ng/ml in the placebo group vs. 492 (2827) ng/ml in the standard AZM group, and 664 (558) ng/ml in the high dose AZM group. Upper and lower bars are maximum and minimum observations.

© 2020 Kong M et al. *JAMA Network Open.*

eFigure 2e: Endotracheal Total MMP-9 Levels

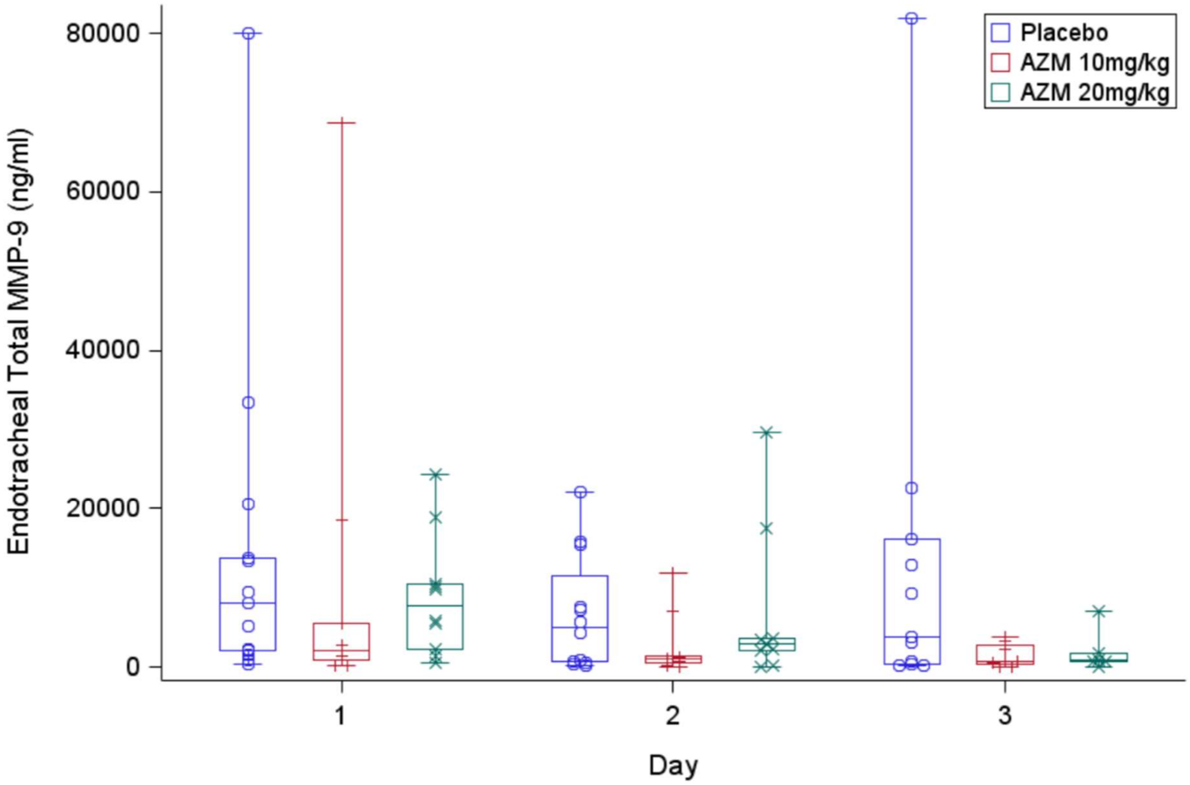

At baseline, the median (IQR) of endotracheal total MMP-9 in the placebo group was 8123 (11697) ng/ml vs. 2079 (4558) ng/ml in the standard AZM group, and 7782 (8286) ng/ml in the high dose AZM group. On day 2, the median (IQR) of endotracheal total MMP-9 was 4976 (10805) ng/ml in the placebo group vs. 998 (855) ng/ml in the standard AZM group, and 2959 (1561) ng/ml in the high dose AZM group. On day 3, the median (IQR) of endotracheal total MMP-9 was 3813 (15713) ng/ml in the placebo group vs. 744 (2474) ng/ml in the standard AZM group, and 873 (1005) ng/ml in the high dose AZM group. Upper and lower bars are maximum and minimum observations.

© 2020 Kong M et al. *JAMA Network Open.*

eFigure 2f: Endotracheal TIMP-1 Levels

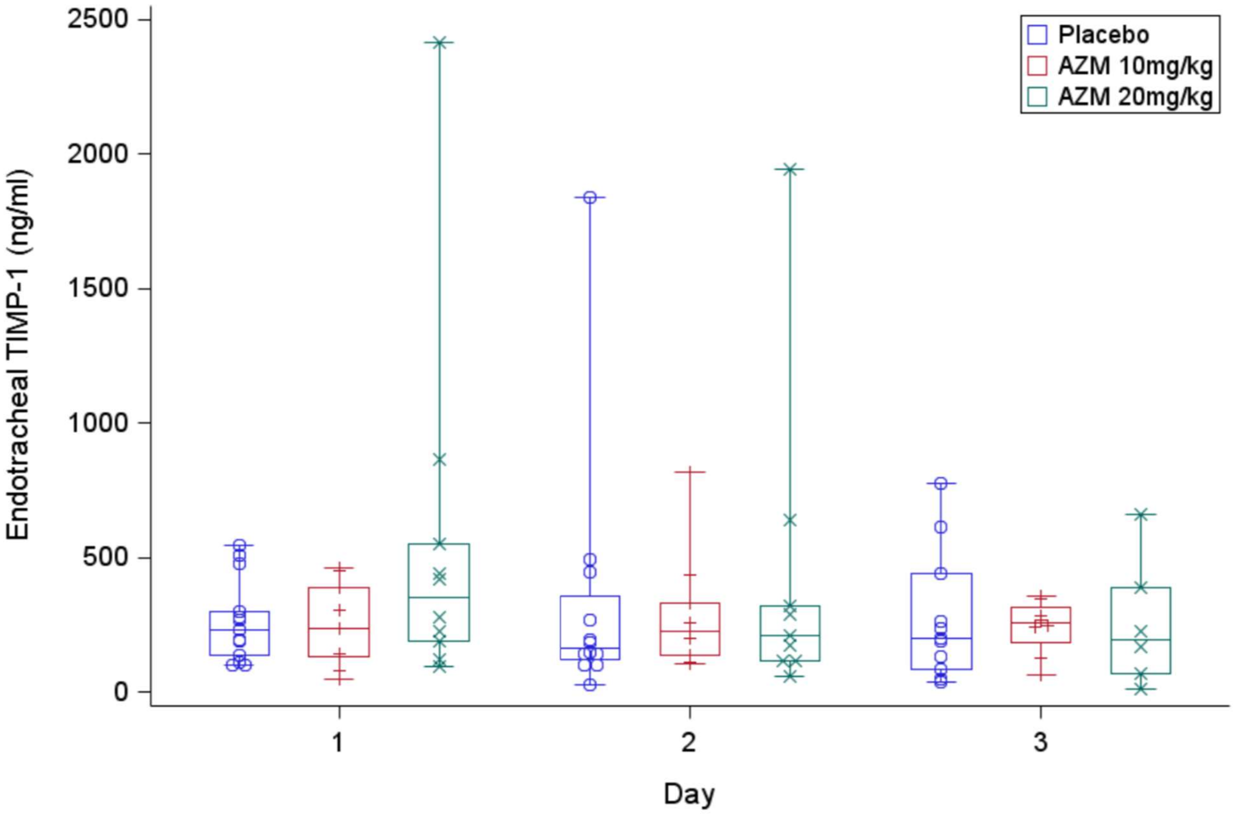

At baseline, the median (IQR) of endotracheal TIMP-1 in the placebo group was 230 (164) ng/ml vs. 350 (359) ng/ml in the standard AZM group, and 237 (255) ng/ml in the high dose AZM group. On day 2, the median (IQR) of endotracheal TIMP-1 was 165 (235) ng/ml in the placebo group vs. 227 (192) ng/ml in the standard AZM group, and 208 (203) ng/ml in the high dose AZM group. On day 3, the median (IQR) of endotracheal TIMP-1 was 199 (353) ng/ml in the placebo group vs. 259 (130) ng/ml in the standard AZM group, and 196 (319) ng/ml in the high dose AZM group. Upper and lower bars are maximum and minimum observations.

© 2020 Kong M et al. *JAMA Network Open.*

eFigure 2g: Endotracheal TNF-α Levels

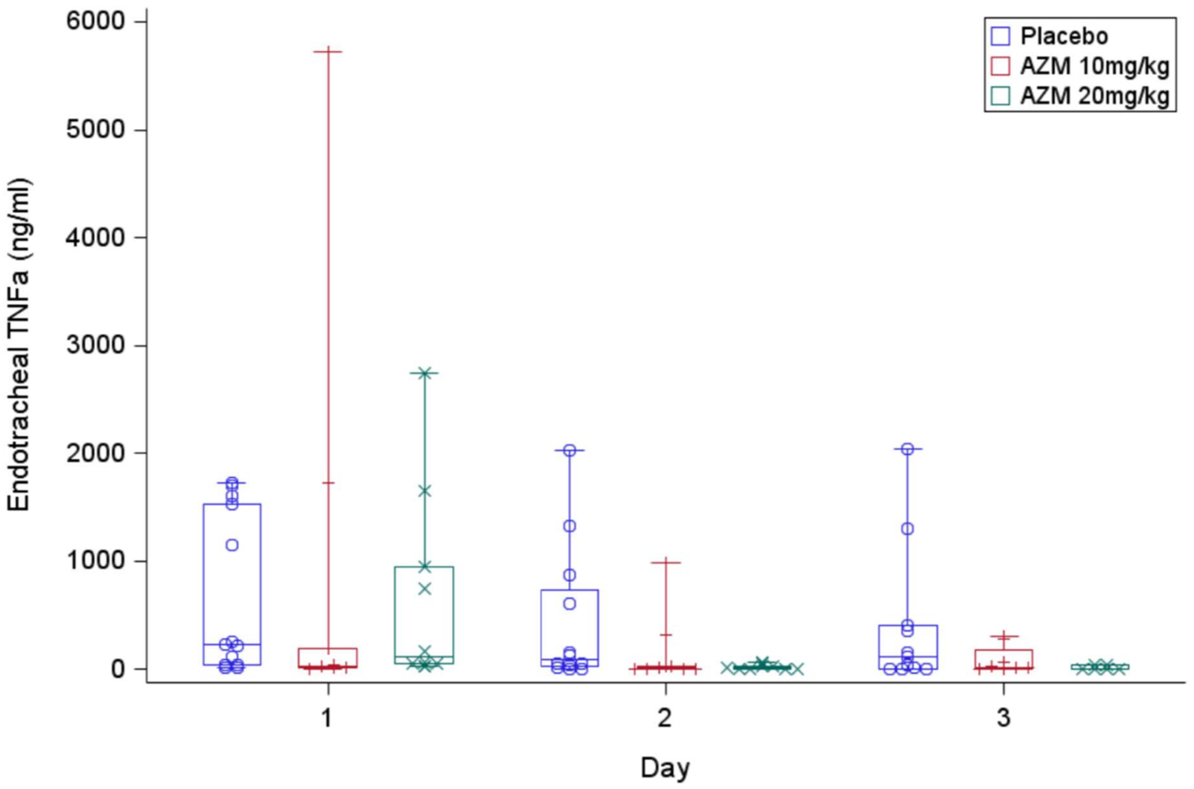

At baseline, the median (IQR) of endotracheal TNF-α in the placebo group was 223 (1488) ng/ml vs. 29 (179) ng/ml in the standard AZM group, and 115 (889) ng/ml in the high dose AZM group. On day 2, the median (IQR) of endotracheal TNF-α was 89 (714) ng/ml in the placebo group vs. 9 (25) ng/ml in the standard AZM group, and 18 (20) ng/ml in the high dose AZM group. On day 3, the mean (SD) of endotracheal TNF-α was 118 (393) ng/ml in the placebo group vs. 17 (165) ng/ml in the standard AZM group, and 4 (30) ng/ml in the high dose AZM group. Upper and lower bars are maximum and minimum observations.

© 2020 Kong M et al. *JAMA Network Open.*

eFigure 2h: Endotracheal IL-1 Levels

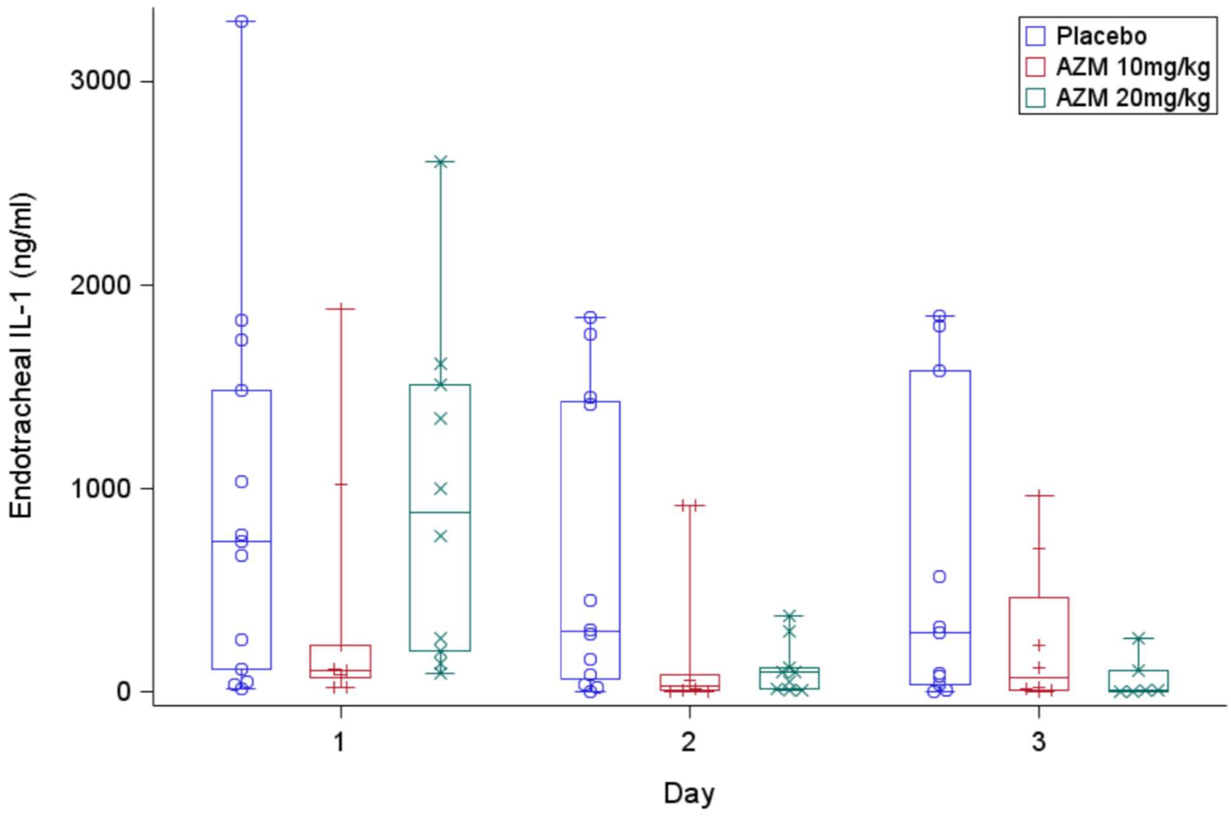

At baseline, the median (IQR) of endotracheal IL-1 in the placebo group was 742 (1373) ng/ml vs. 105 (158) ng/ml in the standard AZM group, and 884 (1309) ng/ml in the high dose AZM group. On day 2, the median (IQR) of endotracheal IL-1 was 294 (1371) ng/ml in the placebo group vs. 25 (72) ng/ml in the standard AZM group, and 94 (108) ng/ml in the high dose AZM group. On day 3, the median (IQR) of endotracheal IL-1 was 292 (1542) ng/ml in the placebo group vs. 73 (455) ng/ml in the standard AZM group, and 8 (104) ng/ml in the high dose AZM group. Upper and lower bars are maximum and minimum observations.

© 2020 Kong M et al. *JAMA Network Open.*

eFigure 2i: Endotracheal IL-10 Levels

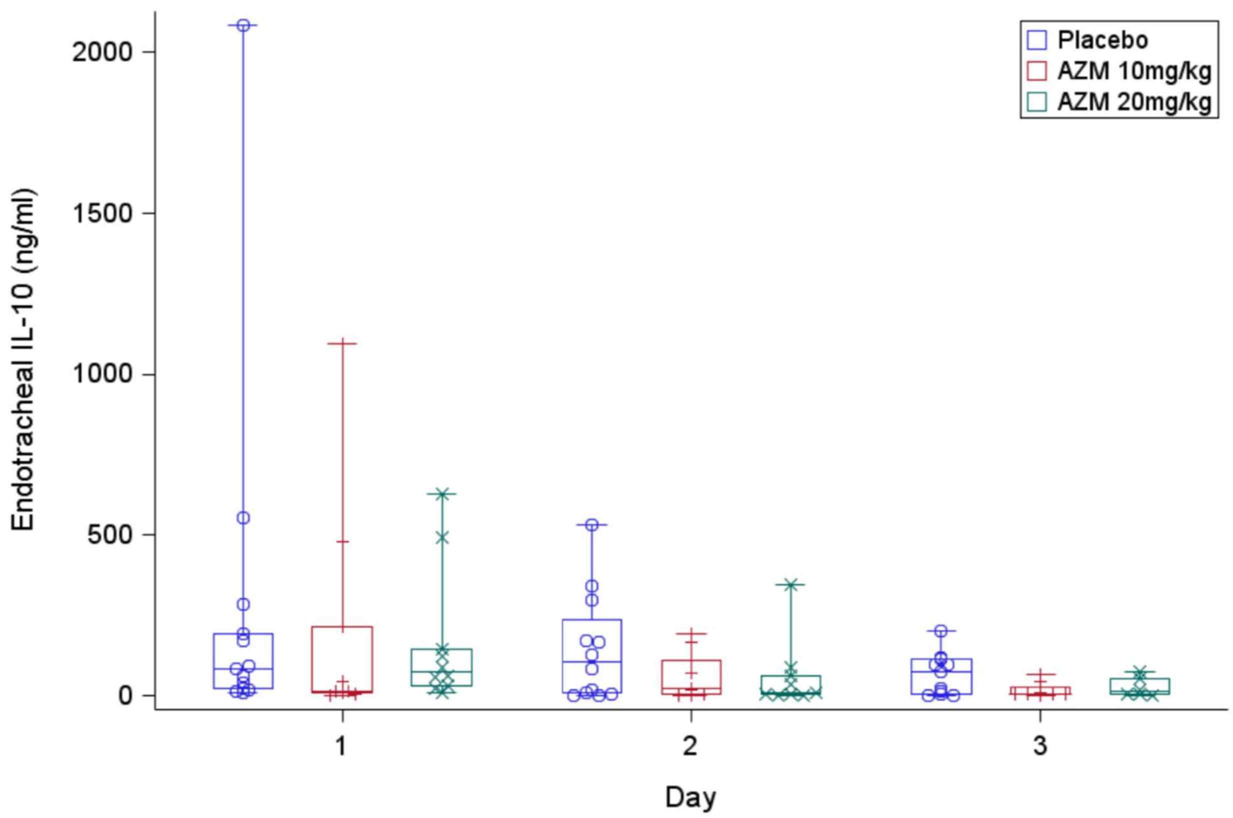

At baseline, the median (IQR) of endotracheal IL-10 in the placebo group was 84 (171) ng/ml vs. 14 (204) ng/ml in the standard AZM group, and 74 (115) ng/ml in the high dose AZM group. On day 2, the median (IQR) of endotracheal IL-10 was 104 (226) ng/ml in the placebo group vs. 24 (104) ng/ml in the standard AZM group, and 9 (60) ng/ml in the high dose AZM group. On day 3, the median (IQR) of endotracheal IL-10 was 74 (108) ng/ml in the placebo group vs. 6 (25) ng/ml in the standard AZM group, and 13 (51) ng/ml in the high dose AZM group. Upper and lower bars are maximum and minimum observations.

© 2020 Kong M et al. *JAMA Network Open.*

eFigure 3. RSV Titer Measured Over Time for Patients in All 3 Groups

eFigure 3a: RSV titer in the nasal compartment decreased over time in all patients

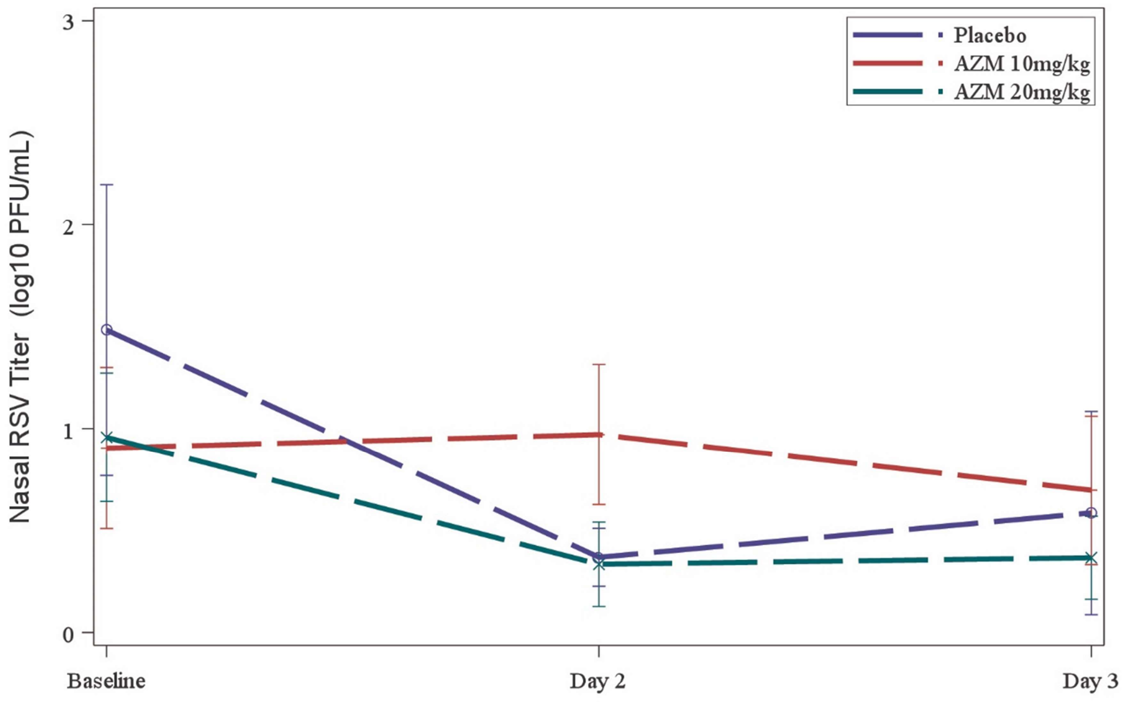

At baseline, the mean RSV load measured in the nasal compartment was similar across all 3 groups at 1.5 ± 2 (log_{10} PFU/mL) in the placebo group vs. 0.9 ± 1.6 (log_{10} PFU/mL) in the standard AZM group vs. 1.0 ± 1.3 (log_{10} PFU/mL) in the high dose AZM group, respectively. On day 2 post-treatment, the RSV load was 0.4 ± 0.6 (log_{10} PFU/mL) in the placebo group vs. 1.0 ± 1.3 (log_{10} PFU/mL) in the standard AZM group vs. 0.3 ± 0.8 (log_{10} PFU/mL) in the high dose AZM. On day 3 post-treatment, the RSV load decreased to 0.6 ± 1.9 (log_{10} PFU/mL) in the placebo group vs. 0.7 ± 1.4 (log_{10} PFU/mL) in the standard AZM group and 0.4 ± 0.8 (log_{10} PFU/mL) in the high dose AZM group, respectively.

© 2020 Kong M et al. *JAMA Network Open.*

eFigure 3b: RSV titer in the endotracheal aspirate decreased over time in all patients

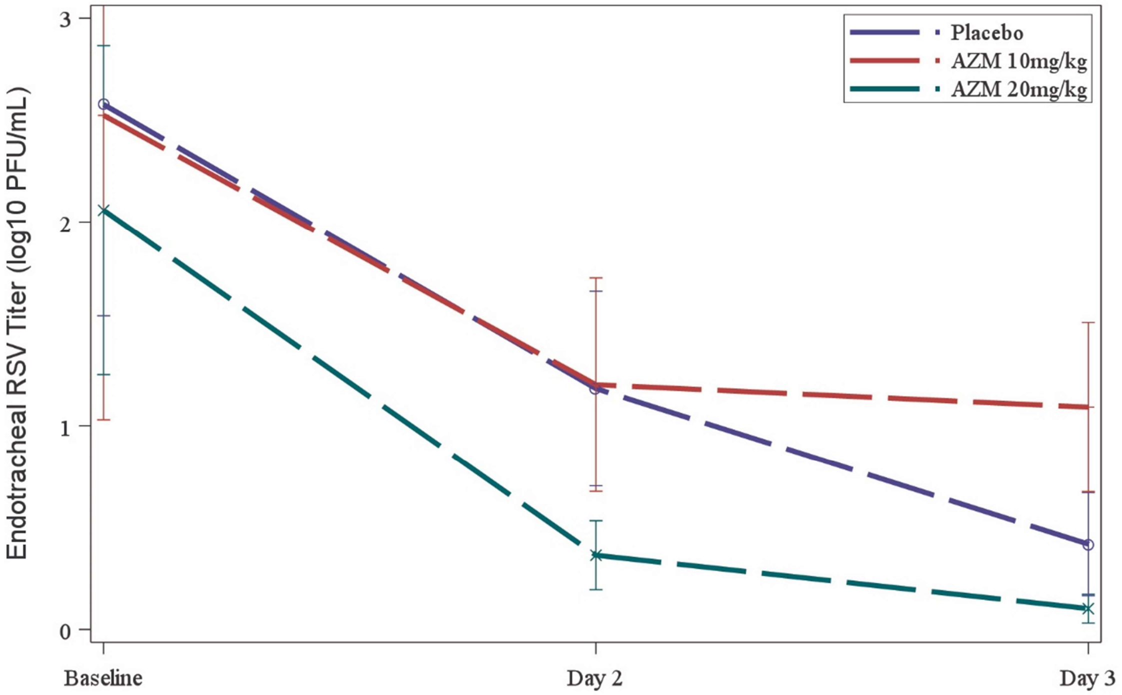

At baseline, the mean RSV load measured in the endotracheal aspirate was no different across all 3 groups at 2.6 ± 1 (log_{10} PFU/mL) in the placebo group vs. 2.5 ± 1.5 (log_{10} PFU/mL) in the standard AZM group vs. 2.1 ± 0.8 (log_{10} PFU/mL) in the high dose AZM group, respectively. On day 2 post-treatment, the RSV load decreased to 1.2 ± 0.48 (log_{10} PFU/mL) in the placebo group vs. 1.2 ± 0.52 (log_{10} PFU/mL) in the standard AZM group vs. 0.36 ± 0.2 (log_{10} PFU/mL) in the high dose AZM. On day 3 post-treatment, the RSV load was lowest in the high dose AZM group at 0.1 ± 0.07 (log_{10} PFU/mL) compared to 1.1 ± 0.42 (log_{10} PFU/mL) in the standard AZM group and 0.42 ± 0.25 (log_{10} PFU/mL) in the placebo group, but this did not reach statistical significance (p=0.49).

© 2020 Kong M et al. *JAMA Network Open.*

eTable. Demographic and Clinical Characteristics at Baseline

Characteristic	No. (%)				*P* value
	Overall (N = 48)	Placebo (n = 16)	AZM		
			10 mg/kg (n = 16)	20 mg/kg (n = 16)	
Age, median (IQR) [range], mo	12.0 (3.0-23.0) [1.0-125.0]	8.0 (2.5-14.5) [1.0, 40.0]	17.5 (3.5-25.0) [1.0-125.0]	9.0 (4.0-28.5) [1.0-79.0]	.36[a]
Weight, median (IQR) [range], kg	8.6 (5.3-11.9) [3.0-23.0]	7.6 (4.6-10.7) [3.0-15.0]	9.6 (6.2-11.9) [4.6-15.0]	6.4 (5.4-14.0) [3.6-23.0]	.39[a]
Height, median (IQR) [range], cm	63.0 (56.0-77.0) [37.0-115.0]	60.0 (52.0-74.0) [44.0-98.0]	66.0 (56.5-76.5) [37.0-111.0]	63.0 (59.0-88.0) [51.0-115.0]	.45[a]
Sex					
Boys	26 (54.2)	9 (56.3)	8 (50.0)	9 (56.3)	.92[b]
Girls	22 (45.8)	7 (43.7)	8 (50.0)	7 (43.7)	
Race					
African American	12 (25.0)	5 (31.3)	5 (31.3)	2 (12.5)	.31[c]
Asian	1 (2.1)	1 (6.2)	0 (0.0)	0	
White and African American	1 (2.1)	0	1 (6.2)	0	
White	34 (70.8)	10 (62.5)	10 (62.5)	14 (87.5)	
Ethnicity					
Hispanic	2 (4.2)	0	1 (6.2)	1 (6.2)	>.99[c]
Non-Hispanic	46 (95.8)	16 (100.0)	15 (93.8)	15 (93.8)	
Comorbidity[d]					
Yes	29 (60.4)	10 (62.5)	9 (56.3)	10 (62.5)	.92[b]
No	19 (39.6)	6 (37.5)	7 (43.7)	6 (37.5)	

Abbreviations: AZM, azithromycin; IQR, interquartile range.

[a]*P* value calculated Kruskal-Wallis test.

[b]*P* value calculated with χ^2 test.

[c]*P* value calculated Fisher exact test.

[d]The most common comorbidity was prematurity (12 [25.0%]) and reactive airway disease (5 [10.4%]). The remaining 12 comorbidities included scoliosis, hemophilia, trisomy 18, trisomy 21, Aicardi syndrome, Townes Brocks syndrome, skeletal dysplasia, seizures, Potter's syndrome, hypoxic ischemic encephalopathy and congenital myopathy.

© 2020 Kong M et al. *JAMA Network Open.*
