# Supplementary material for: Azithromycin Treatment vs Placebo in Children With Respiratory Syncytial Virus–Induced Respiratory Failure: A Phase 2 Randomized Clinical Trial
Source: JAMA Netw Open. 2020 Apr 23;3(4):e203482. doi: 10.1001/jamanetworkopen.2020.3482 (PMC7180420; doi:10.1001/jamanetworkopen.2020.3482)
Supplement: Supplement 3. — Data Sharing Statement [file jamanetwopen-3-e203482-s003.pdf]

Data Sharing Statement

Kong. Azithromycin Treatment vs Placebo in Children With Respiratory Syncytial Virus-Induced Respiratory Failure. *JAMA Netw Open*. Published April 23, 2020. 10.1001/jamanetworkopen.2020.3482

Data
Data available: Yes
Data types: Deidentified participant data
How to access data: Request to be sent to the PI; mkong@peds.uab.edu
When available: With publication

Supporting Documents
Document types: None

Additional Information
Who can access the data: Researchers whose proposed use of data has been approved
Types of analyses: For any purpose
Mechanisms of data availability: with a signed data access agreement
